# Differential pathology and susceptibility to MBNL loss across muscles in myotonic dystrophy mouse models

**DOI:** 10.1172/jci.insight.195836

**Published:** 2025-08-14

**Authors:** Mackenzie L. Davenport, Amaya Fong, Gloria Montoya-Vazquez, Maria Fernanda Alves de Moura, Jodi L. Bubenik, Maurice S. Swanson

**Affiliations:** 1 Department of Molecular Genetics and Microbiology,; 2 Center for NeuroGenetics,; 3 UF Genetics Institute, and; 4 Myology Institute, University of Florida, Gainesville, Florida, USA.

**Keywords:** Genetics, Muscle biology, Genetic diseases, Mouse models, Muscle

## Abstract

There are 2 subtypes of myotonic dystrophy, DM1 and DM2, each caused by repeat expansion mutations. The leading pathogenic mechanism is RNA-mediated toxicity, whereby (C)CUG expansions sequester the muscleblind-like (MBNL) family of RNA binding proteins. However, key differences exist in muscle involvement patterns and histopathology between DM1 and DM2. The cause of these disparities both in how the muscles are affected within each disease and between the 2 diseases is unknown, and it is unclear if current DM mouse models recapitulate these differences or develop differential muscle susceptibility. Here, we examined the expression of disease-relevant genes across healthy human muscles from a transcriptomic atlas and collected a series of muscles from *Mbnl*-KO mice to evaluate characteristic histologic and molecular features of DM pathology. Our results indicate that MBNL loss discordantly affects muscles, likely through a splicing-independent mechanism, and results in a fiber atrophy profile more like DM1 than DM2. These findings point to a predominant role for MBNL loss in muscle pattern involvement in DM1, provide further evidence for additional DM2 pathomechanisms, and have important implications for muscle choice when performing analyses in new mouse models and evaluating therapeutic modalities and biomarkers.

## Abstract 2

Muscles in myotonic dystrophy mouse models display differential susceptibility to MBNL loss with implications for muscle choice in testing outcome measures.

## Introduction

**Figure 1:**
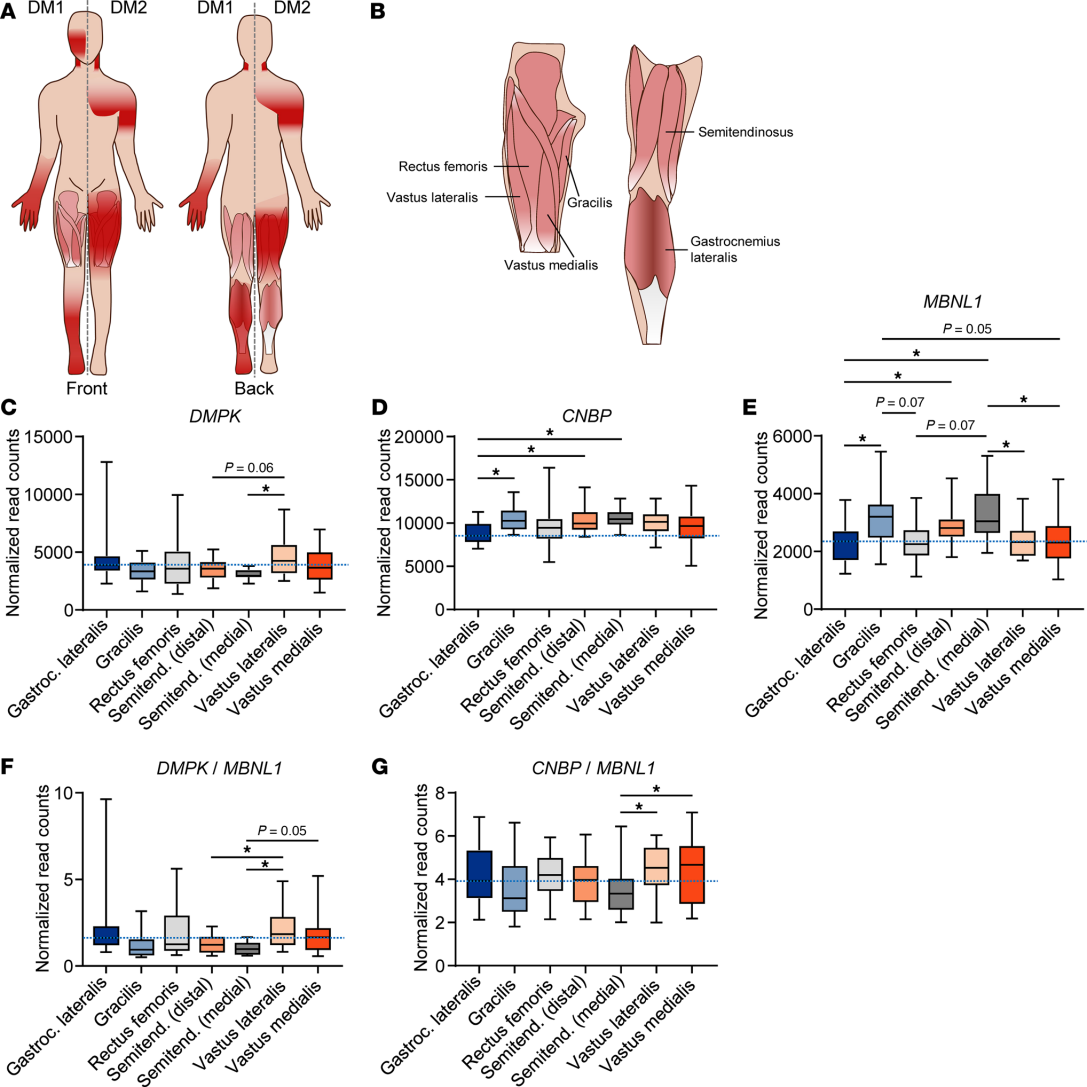
Differential disease relevant gene expression across healthy human muscles. (**A**) Muscles affected in DM1 and DM2. Dark red shading indicates muscle groups more highly affected in each disease. Figure adapted from Wenninger et al. *Front Neurol*. 2018. (**B**) Diagram depicting human muscles biopsied by Abbassi-Daloii et al. (17). Paired samples from 20 healthy males 25 ± 3.6 years old. (**C**–**E**) Normalized read counts of RNA-Seq data from Abbassi-Daloii et al. (17) for *DMPK* (**C**), *CNBP* (**D**), and *MBNL1* (**E**). (**F**) Ratio of *DMPK*/*MBNL1* normalized read counts. (**G**) Ratio of *CNBP*/*MBNL1* normalized read counts. *P < 0.05; 1-way ANOVA with Tukey’s multiple-comparison test. Dotted lines indicate mean expression in the gastrocnemius lateralis, the only distal muscle biopsied for analysis.

Each of the human muscular dystrophies develops a unique pattern of muscle involvement, where some muscles are affected earlier or more severely than others. For example, limb girdle muscular dystrophy (LGMD) is so named for the early involvement and weakness of the proximal muscles of the shoulder and pelvic girdles (1), while facioscapulohumeral muscular dystrophy (FSHD) is named for the characteristic weakness and atrophy of face, shoulder girdle, and upper arm muscles (2). The myotonic dystrophies are no different and present with distinct patterns of muscle involvement, although these patterns differ between the subtypes of myotonic dystrophy (Figure 1A) (3). Myotonic dystrophy type 1 (DM1), caused by a CTG repeat expansion mutation in the 3′UTR of the *DMPK* gene, presents primarily as a distal myopathy, affecting the face, neck, and distal limb muscles. In contrast, DM2, caused by a CCTG repeat expansion in intron 1 of the *CNBP* (formerly *ZNF9*) gene, presents primarily as a proximal myopathy, affecting the neck and proximal muscles of the limbs, including the shoulders and thighs. Most muscular dystrophies ultimately result in fatty and fibrotic replacement of the muscle, and MRI of affected patients with DM1 shows the greatest muscle fat fraction in the distal lower limb muscles, particularly the gastrocnemius medialis, soleus, and tibialis anterior (TA) muscles with sparing of the proximal rectus femoris muscle (4–7). This is opposed to MRI findings in DM2, which typically have less severe fat infiltration that appears more diffuse rather than selective and primarily affects the posterior muscles of the thigh (4, 5, 8). While atrophic fibers, pyknotic nuclear clumps, basophilic regenerating fibers, fibrosis, adipose accumulation, and centralized myonuclei are hallmark histopathological features of both diseases, there are also histological differences in how the muscles are affected between DM1 and DM2 (9). DM1 results in both a predominance and selective atrophy of Type I myofibers with some hypertrophy of Type II myofibers (9, 10), and Type I fibers are selectively centronucleated. In contrast, DM2 is characterized by a selective atrophy of Type II myofibers which are selectively centronucleated (11, 12). An open question in the field is why these 2 diseases present disparately despite a shared pathomechanism whereby the (C)CUG repeat expansion RNAs sequester the muscleblind-like (MBNL) family of RNA-binding proteins (RBPs), leading to broad spliceopathy (13–15). One hypothesis is that the expression of the host genes (*DMPK*, *CNBP*) differs among different muscles, leading to a higher pathogenic repeat load across different muscles. Another hypothesis is that additional loss of DMPK or CNBP protein, or haploinsufficiency, contributes to disease pathology. A third hypothesis is that the DM2 CCUG repeats sequester additional RNA binding proteins such as the RBFOX family that are not affected in DM1 (16). Yet another hypothesis is that there are simply intrinsic differences across the muscles that contribute to their differences in shortening velocity, resistance to fatigue, and innervation, which also reflect how they differentially respond to disease (17).

There has not been a concerted effort to determine if DM mouse models recapitulate the pattern of muscle involvement and differential muscle susceptibility both within each disease as well as between the 2 DM types. A recent study performed a meta-analysis of publicly available RNA-Seq data to assess alternative splicing changes across the gastrocnemius, quadriceps, and TA muscles of HSA^LR^ mice, which is a transgenic DM1 mouse model that expresses a human skeletal actin transgene with ~250 CTG long repeats in the 3′UTR (18, 19). This study found the highest levels of transgene expression in the gastrocnemius followed by the quadriceps with the lowest levels in the TA, and transgene expression correlated with the extent of splicing dysregulation (19). Another study evaluated the susceptibility of different HSA^LR^ muscles (extensor digitorum longus [EDL], soleus, diaphragm) to myotonia and force impairment. This study found myotonia and impaired force production only in the EDL muscle, with no evidence of myotonia or impaired force in the soleus or diaphragm muscles (20). While these results are important for work utilizing the HSA^LR^ model and indicate differential muscle pathology in mice, they are difficult to translate to other models due to potential transgene integration site effects and overexpression of human skeletal actin. HSA^LR^ mice also do not allow us to directly examine the contributions of MBNL loss to differential pathology.

To address why these 2 diseases present disparate muscle phenotypes despite shared sequestration of MBNL proteins, we determined the expression of *DMPK*, *CNBP*, and *MBNL1* genes across healthy human muscles using a transcriptomic atlas, and we evaluated multiple muscles from *Mbnl*-KO mice to assess differential susceptibility, which muscles better recapitulate DM pathology, and whether mouse muscles resemble DM1, DM2, or neither following MBNL loss.

## Results

### Differential disease-relevant gene expression across healthy human muscles.

One obvious explanation for the differential sensitivity of human muscles to DM pathology would be higher expression of pathogenic repeat–containing genes (e.g., *DMPK,* *CNBP*) or lower expression of sequestered RBPs (e.g., MBNL1) in more severely affected muscles, leading to more complete sequestration and less free MBNL (21). This hypothesis proves challenging to test, as obtaining biopsies of multiple muscles from a single individual is generally unrealistic; however, a recent study created a transcriptomic atlas of several leg muscles (gastrocnemius lateralis, vastus lateralis, vastus medialis, rectus femoris, semitendinosus [biopsied medially and distally], and gracilis) from healthy volunteers (Figure 1B) (17). While the only distal or lower leg muscle included in this study is the gastrocnemius lateralis and, thus, it does not include many of the distal muscles highly affected in DM1, it does provide a starting place to examine the expression of genes implicated in DM pathology across human muscles. There is not much variation in *DMPK* expression across the muscles biopsied, although there is significantly less *DMPK* in the semitendinosus than the vastus lateralis (Figure 1C). There is a clearer delineation in *CNBP* expression between the distal gastrocnemius lateralis and each of the proximal muscles. *CNBP* expression is lower in the typically less-affected (in DM2) distal gastrocnemius muscle than the more-affected proximal muscles, with little variation in *CNBP* expression among the proximal muscles (Figure 1D). There is considerably more variation in the expression of *MBNL1* (the most highly expressed MBNL paralog in skeletal muscle) across muscles, with the lowest expression in the gastrocnemius lateralis and each of the 3 quadriceps muscles biopsied: the rectus femoris, vastus lateralis, and vastus medialis (Figure 1E). The expression of *MBNL2*, though less abundant in skeletal muscle, follows a similar pattern across the muscles with the least *MBNL2* expression in the gastrocnemius lateralis (Supplemental Figure 1A; supplemental material available online with this article; https://doi.org/10.1172/jci.insight.195836DS1). Perhaps a greater predictor of muscle susceptibility to DM pathogenesis, however, is the ratio of *DMPK*/*MBNL1* and *CNBP*/*MBNL1*, which should more accurately predict the MBNL sequestration potential of the pathogenic repeats. We therefore examined both of these ratios within each subject’s muscles. While there still isn’t extensive variation of the *DMPK/MBNL1* ratio among muscles, the gastrocnemius lateralis, vastus lateralis, and vastus medialis have the highest ratio of *DMPK*/*MBNL1* (Figure 1F). Comparing these ratios to published MRI data quantifying fat accumulation in the lower limb muscles of patients with DM1 (7), we find that, out of the 6 muscles for which we have RNA-Seq data from healthy patients, the greatest degree of fat infiltration in DM1 muscle is in the gastrocnemius lateralis, vastus lateralis, and vastus medialis, with lower fat infiltration in the semitendinosus, rectus femoris, and gracilis (7). This degree of fat infiltration within the muscles correlates with the ratio of *DMPK*/*MBNL1*. Examining the ratio of *CNBP*/*MBNL1* introduces more variability in the data than just examining *CNBP* expression, although the muscles with the greatest ratio of *CNBP*/*MBNL1* are the muscles of the quadriceps (rectus femoris, vastus lateralis, vastus medialis) (Figure 1G). The ratio of *CNBP*/*MBNL1* in the context of DM2 may also be less useful than *DMPK*/*MBNL1* in DM1 since additional RBPs such as RBFOX1/2 have been reported as additionally sequestered by the CCUG repeats in DM2 (16). The expression of both *RBFOX1* and *RBFOX2*, however, follows a similar pattern to the *MBNL* genes with the lowest expression in the gastrocnemius lateralis (Supplemental Figure 1, B and C). We also examined the expression of *CELF1* (formerly *CUGBP1*) and *HNRNPA1*, which have been implicated in DM1 pathogenesis (22–25), and we found much greater variation in *CELF1* expression among individuals than any of the other genes examined (Supplemental Figure 1D), while *HNRNPA1* showed little variation both between individuals and across muscles (Supplemental Figure 1E). To summarize, the ratio of *DMPK/MBNL1* correlates with the fat fraction composition of DM1 muscle, largely driven by differences in *MBNL1* expression across muscles, while the level of *CNBP* alone correlates with muscle involvement in DM2.

### Differential disease relevant gene expression across WT mouse muscles.

**Figure 2:**
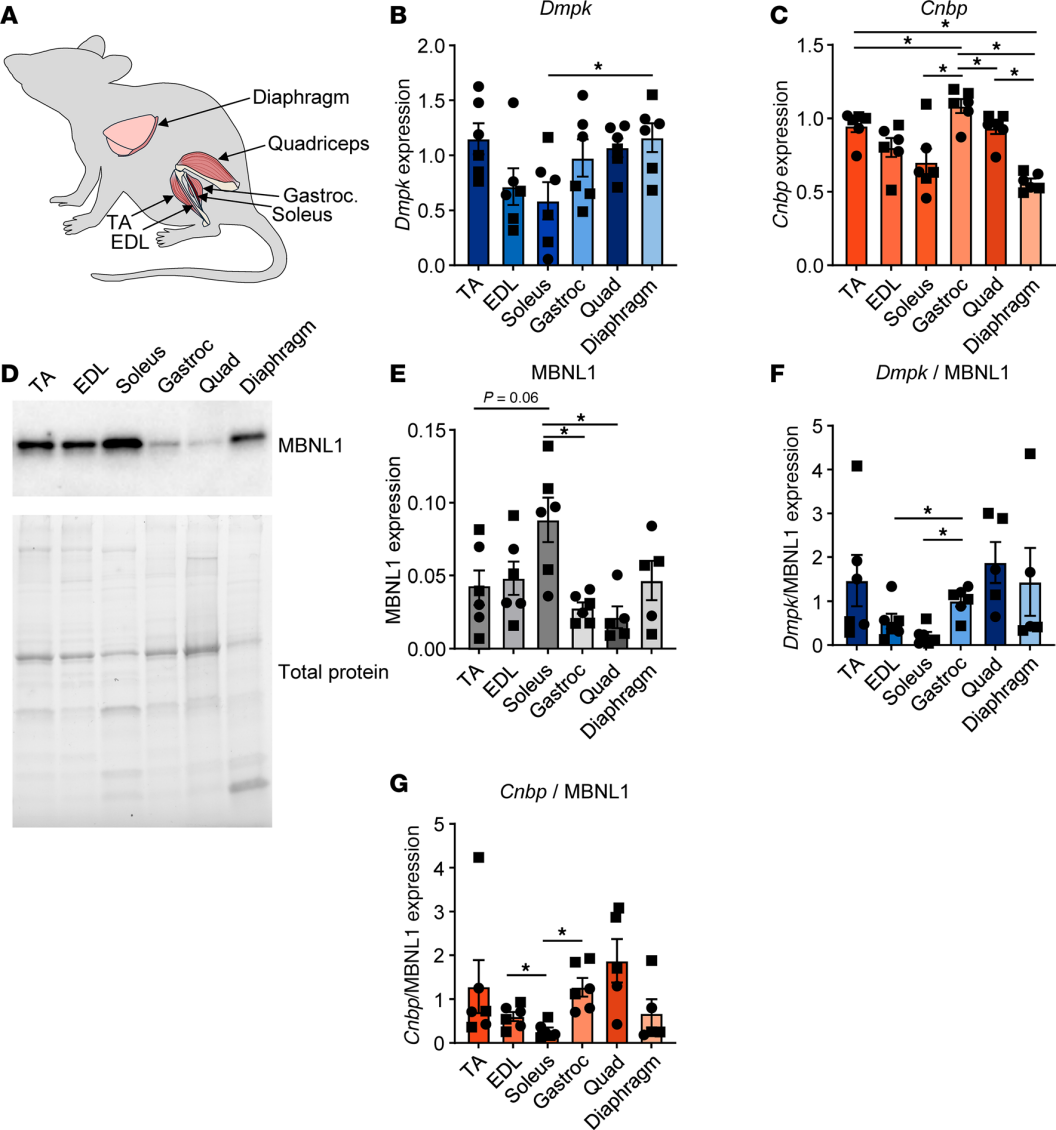
Differential disease relevant gene expression across WT mouse muscles. (**A**) Diagram depicting mouse muscles examined. (**B** and **C**) Mean *Dmpk* (**B**) and *Cnbp* (**C**) mRNA expression determined by qPCR in 8- to 12-week-old WT FVB mouse muscles (data are shown as ± SEM: *P < 0.05; 1-way ANOVA with Tukey’s multiple-comparison test). Expression normalized to geometric mean of 3 reference genes. *n* = 6 mice. (**D**) Western blot for MBNL1 in 8- to 12-week-old WT FVB mouse muscles. Total protein determined by Bio-Rad stain-free technology. Data are representative of 6 independent experiments. (**E**) Quantification of MBNL1 Western blots (data are shown as ± SEM: *P < 0.05; 1-way ANOVA with Tukey’s multiple-comparison test). Expression normalized to total protein. (**F** and **G**) Ratio of *Dmpk* (**F**) and *Cnbp* (**G**) mRNA to MBNL1 protein in each mouse muscle (data are shown as mean ± SEM: *P < 0.05; 1-way ANOVA with Tukey’s multiple-comparison test). Squares indicate males and circles indicate females.

Since most DM therapeutics in development are tested in mouse models, it is important to determine if mouse muscles also have varying levels of disease relevant gene expression and whether mouse muscles are differentially susceptible to pathogenesis. We began by examining the expression of *Dmpk* RNA, *Cnbp* RNA, and MBNL1 protein across some of the most commonly collected mouse muscles: TA, EDL, soleus, gastrocnemius, quadriceps, and diaphragm (Figure 2A). While there is considerable variation in *Dmpk* expression among animals, there is generally less *Dmpk* in the soleus than any other muscles tested followed by the EDL (Figure 2B). In several muscles such as the TA, gastrocnemius, and quadriceps, females also tend to have higher levels of *Dmpk* expression than males. Similarly, the soleus also has lower levels of *Cnbp* compared with most other muscles except the diaphragm, which also has lower *Cnbp* expression (Figure 2C). We did not detect sex differences in *Cnbp* expression. Because it is the MBNL1 protein that is primarily sequestered by the pathogenic RNA repeats in DM muscle, we evaluated MBNL1 protein levels across the mouse muscles (Figure 2, D and E). The soleus has significantly higher levels of MBNL1 compared with the other muscles, while the muscles with the lowest expression of MBNL1 are the gastrocnemius and quadriceps (Figure 2, D and E). Accordingly, the muscles with the highest ratio of *Dmpk*/MBNL1 are the TA and quadriceps, while the soleus has a significantly lower ratio (Figure 2F). The ratio of *Cnbp*/MBNL1 is similar, with the TA and quadriceps having the highest ratio, and the soleus, EDL, and diaphragm having lower ratios of *Cnbp*/MBNL1 (Figure 2G). We also evaluated the expression of additional RBPs implicated in DM pathogenesis, including CELF1, RBFOX1, and HNRNPA1 (Supplemental Figure 2, A–D) (16, 22–25). There were minimal differences in the expression of CELF1 and RBFOX1 across mouse muscles, although generally the expression in the TA was lowest with highest expression in the diaphragm for both proteins (Supplemental Figure 2, A–C). There was greater variability in HNRNPA1 expression with the highest expression in the EDL followed by the soleus (Supplemental Figure 2, A and D). We would, thus, expect that in an ideal mouse model of DM1 or DM2 expressing (C)CTG repeat expansions in their respective endogenous loci that the TA and quadriceps muscles would be most affected, while the soleus muscle would be least affected. While several labs are attempting to develop such models, there are not currently phenotypic models available that meet these criteria. Therefore, to address which muscles might be most affected by DM pathogenesis in the mouse and because we observed the greatest variation in *MBNL* expression in human muscles, we evaluated each of these muscles in *Mbnl*-KO mice. Because MBNL2 is upregulated when MBNL1 is knocked out and, thus, compensates for some of its function (26), it is important to also knock down or knock out MBNL2. The greatest constitutive KO of MBNL compatible with postnatal life is *Mbnl1^–/–^ Mbnl2^+/–^* (26). We therefore evaluated both *Mbnl1^–/–^* and *Mbnl1^–/–^ Mbnl2^+/–^* mice. Evaluating *Mbnl*-KO mice also allowed us to examine whether loss of MBNL reflects a more DM1- or DM2-like histopathology.

### Susceptibility of mouse muscles to mass loss or hypertrophy following MBNL loss.

**Figure 3:**
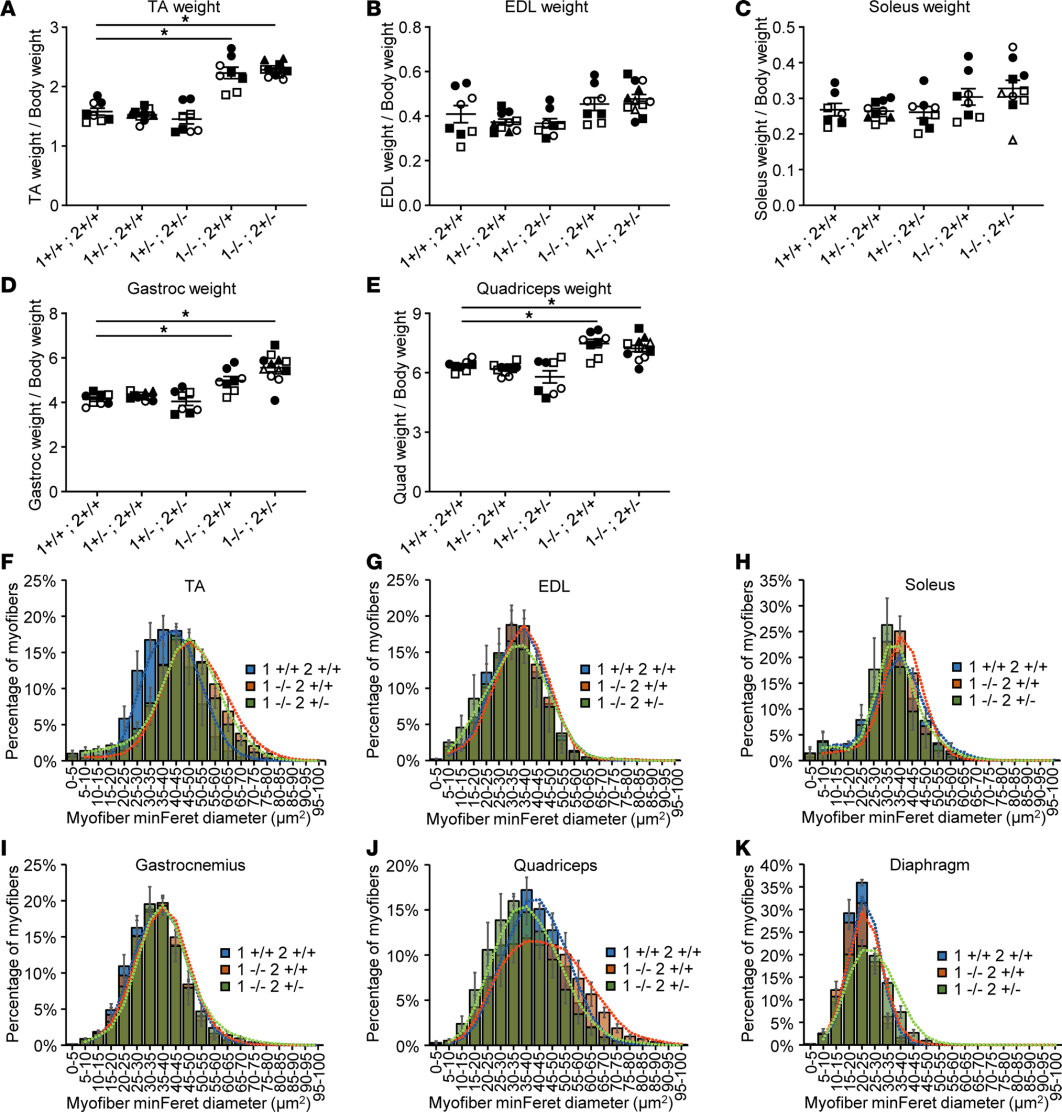
Mouse muscles display increased mass and hypertrophy with MBNL loss. (**A**–**E**) Mean muscle weight normalized to body weight (mg/g) across an allelic series of 8-week-old *Mbnl*-KO mice (data are shown as mean ± SEM: *P < 0.05; 1-way ANOVA with Dunnett’s multiple-comparison test). 1 = *Mbnl1*, 2 = *Mbnl2*. Distinct shapes indicate different animals. Consistent filled or unfilled shapes indicate muscles from the same animal. *n* = 4–6 male mice per genotype. (**F**–**K**) Myofiber size distribution by MinFeret diameter of the indicated muscles from WT, *Mbnl1*^–/–^, and *Mbnl1^–/–^ Mbnl2^+/–^* mice (data are shown as mean ± SEM). Bars are overlapping. *n* = 3 male mice per genotype.

While muscle atrophy is a hallmark symptom of DM1 and a late symptom of DM2, calf hypertrophy is an early symptom in DM2 (27, 28). We, thus, first assessed whether the muscles of our *Mbnl*-KO mice displayed signs of atrophy or hypertrophy by weighing the muscles at collection. All muscles were collected at 8 weeks of age, as *Mbnl1^–/–^ Mbnl2^+/–^* FVB/NJ mice rapidly die or reach humane endpoint at this time. Body weight of *Mbnl1^–/–^* mice is not significantly different than WT, but *Mbnl1^–/–^ Mbnl2^+/–^* mice are significantly smaller than their WT littermates (Supplemental Figure 3, A and B). Therefore, to better assess the possibility of muscle atrophy or hypertrophy, muscle weights were normalized to total body weight. Unnormalized muscle masses are shown in Supplemental Figure 3. When normalized, TA muscles from both *Mbnl1^–/–^* and *Mbnl1^–/–^ Mbnl2^+/–^* mice are bigger than their WT littermate controls in both male and female mice (Figure 3A and Supplemental Figure 4A). While EDL and soleus muscles also trend bigger in male *Mbnl1^–/–^* and *Mbnl1^–/–^ Mbnl2^+/–^* mice, this difference reaches statistical significance in females (Figure 3, B and C, and Supplemental Figure 4, B and C). The gastrocnemius and quadriceps muscles are also bigger in male *Mbnl1^–/–^* and *Mbnl1^–/–^ Mbnl2^+/–^* mice, but only the quadriceps in female *Mbnl1^–/–^* mice trends higher as well (Figure 3, D and E, and Supplemental Figure 4, D and E).

To determine if this increase in muscle size is due to myofiber hypertrophy, we performed myofiber size distribution analysis on male *Mbnl1^–/–^* and *Mbnl1^–/–^ Mbnl2^+/–^* mice and their WT littermates. TA muscles from *Mbnl1^–/–^* and *Mbnl1^–/–^ Mbnl2^+/–^* display clear hypertrophy by the rightward shift of their myofiber size distribution curves (Figure 3F). There is no change in the myofiber size distribution curves of the EDL, soleus, or gastrocnemius muscles (Figure 3, G–I). Quadriceps muscles from *Mbnl1^–/–^* mice also have a rightward shift of their myofiber size distribution curve, although to a lesser extent than the TA muscle, while in *Mbnl1^–/–^ Mbnl2^+/–^* mice, the curve is shifted slightly left, indicating possible atrophy (Figure 3J). In the diaphragm, no hypertrophy is noted in *Mbnl1^–/–^* mice, but there is a rightward shift of the myofiber size distribution curve in *Mbnl1^–/–^ Mbnl2^+/–^* mice, indicating some hypertrophy, resulting in thickening of the diaphragm muscle (Figure 3K and Supplemental Figure 5). These results initially indicated that the TA and quadriceps muscles might be more susceptible to MBNL loss.

### The TA muscle recapitulates more DM-like histopathology than other muscles.

**Figure 4:**
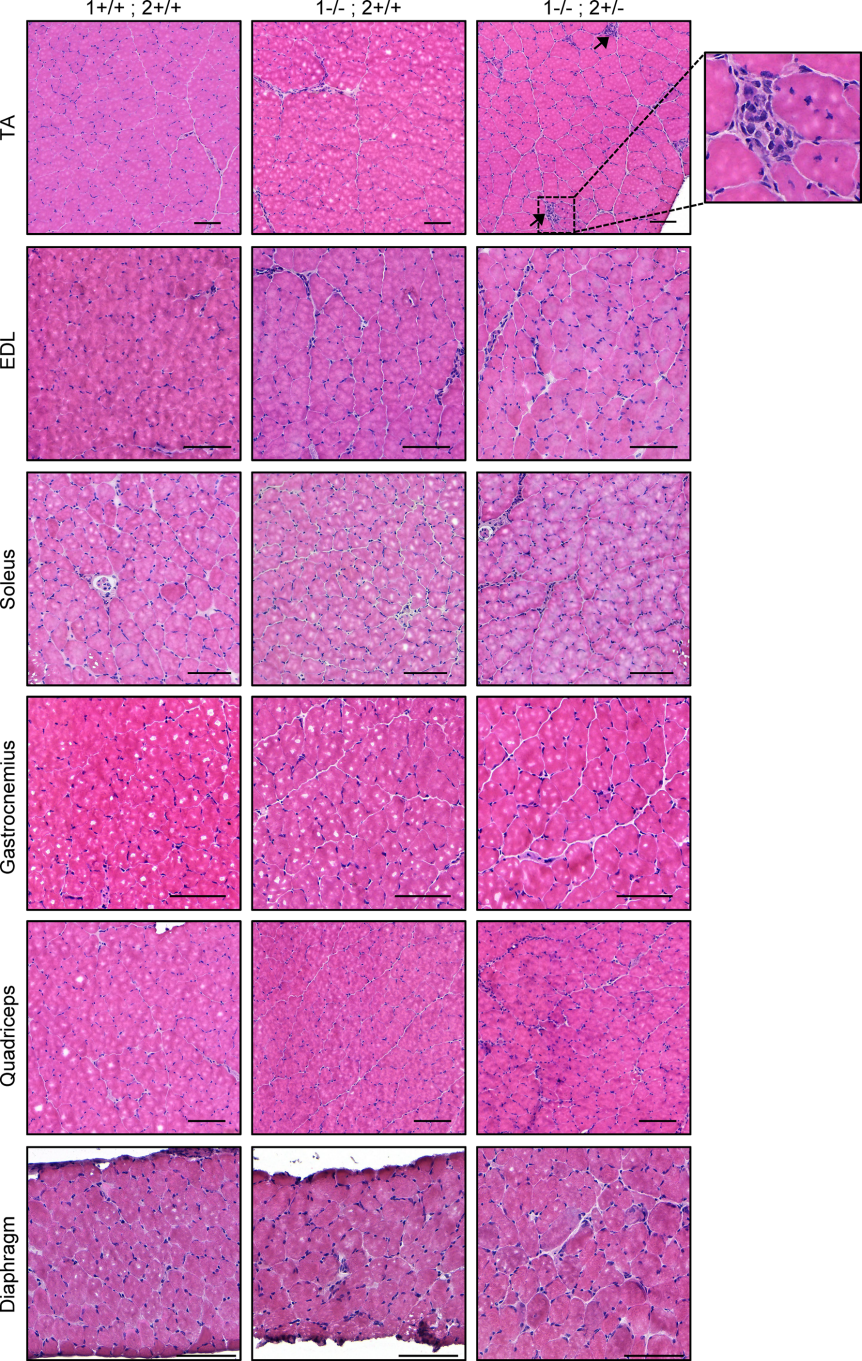
The TA muscle recapitulates more DM-like histopathology than other muscles. H&E staining of TA, EDL, soleus, gastrocnemius, quadriceps, and diaphragm muscles from 8-week-old WT, *Mbnl1*^–/–^, and *Mbnl1^–/–^ Mbnl2^+/–^* mice. Scale bar: 100 μm. Arrows indicate severely atrophic fibers with clustered nuclei. *n* = 4 mice per genotype. Note: the presence of white dots/circles in the center of myofibers is freeze artifact and does not reflect pathology. This does not influence the interpretation of results.

**Figure 5:**
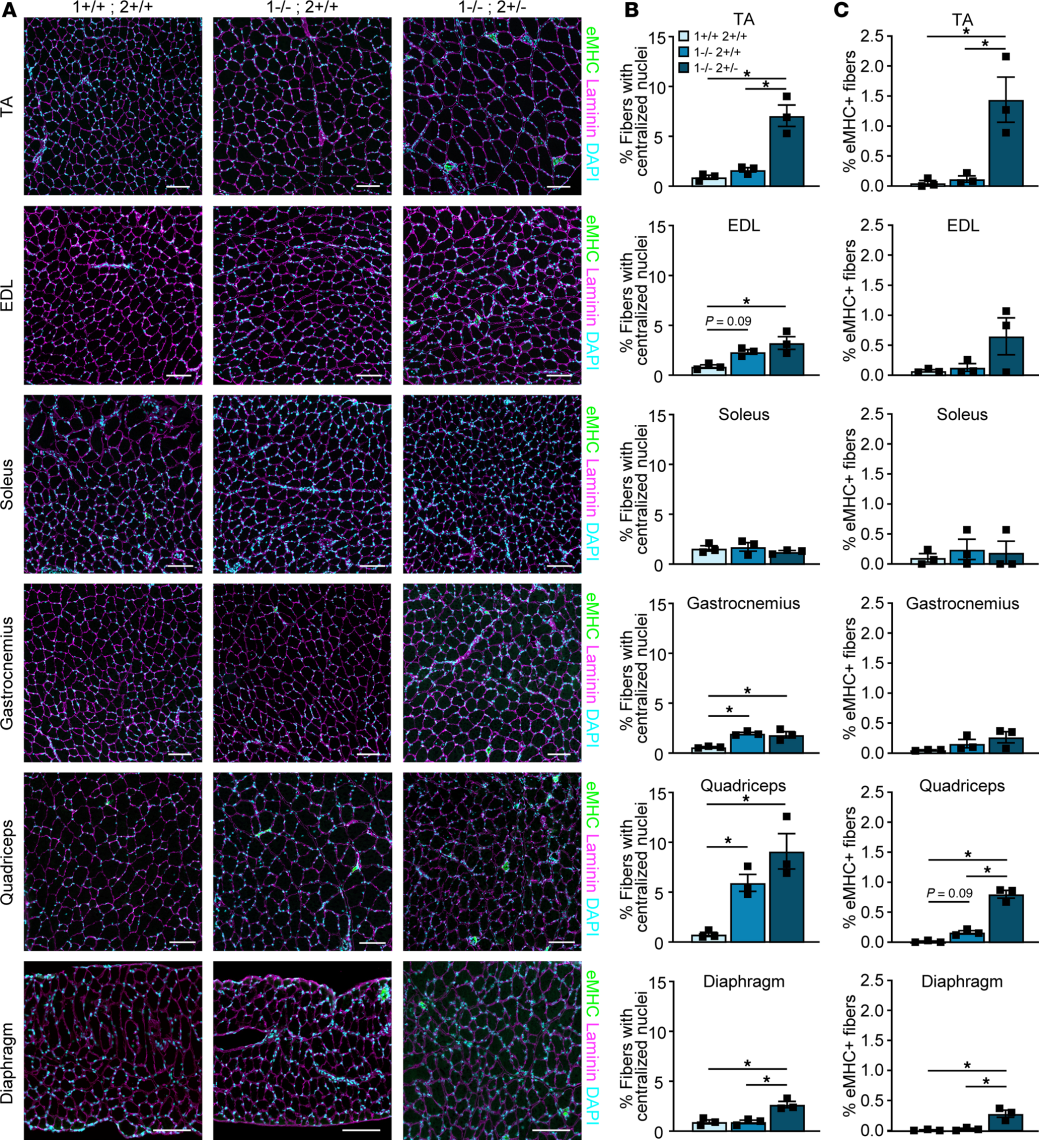
The TA and quadriceps muscles develop the greatest percentage of centrally nucleated and eMHC^+^ myofibers. (**A**) Immunofluorescence of eMHC, Laminin, and DAPI in muscles from 8-week-old WT, *Mbnl1*^–/–^, and *Mbnl1^–/–^ Mbnl2^+/–^* mice. Scale bar: 100 μM. (**B**) Quantification of percent myofibers with centralized nuclei per cross-section (data are shown as mean ± SEM, *P < 0.05, 1-way ANOVA with Tukey’s multiple-comparison test). *n* = 3 mice per genotype. (**C**) Quantification of percent eMHC^+^ myofibers per cross-section (data are shown as mean ± SEM, *P < 0.05, 1-way ANOVA with Tukey’s multiple-comparison test).

To determine if any muscle developed more overt signs of DM-like histopathology (such as centralized myonuclei, pyknotic nuclear clumps, or atrophic fibers), we stained each of the muscles for H&E (Figure 4 and Supplemental Figure 5). There was minimal pathology noted in the muscles of *Mbnl1^–/–^* mice, aside from some myofiber size variability and the occasional centronucleated myofiber. In contrast, in the TA muscle of *Mbnl1^–/–^ Mbnl2^+/–^* mice, we observed many more signs of DM-related pathology, including what appeared to be a greater number of centronucleated myofibers, atrophic fibers, and severely atrophic fibers with clustered nuclei. This mirrors what has previously been observed (26); however, we did not observe such severely atrophic cluster-nucleated fibers in any other muscle examined. To quantify the percentage of centronucleated myofibers, we stained each muscle with laminin and DAPI (Figure 5A and Supplemental Figure 6). Most muscles from *Mbnl1^–/–^* mice displayed a small increase in the percentage of centronucleated myofibers, except for the soleus and diaphragm, which did not show any increase in centrally nucleated fibers in *Mbnl1^–/–^* compared with WT muscle (Figure 5B). An even greater percentage of centrally nucleated fibers was observed in muscles from *Mbnl1^–/–^ Mbnl2^+/–^* mice, with the TA and quadriceps having the greatest percentage of centronucleated myofibers (Figure 5B). Notably, the soleus still did not have any increase in centrally nucleated myofibers in *Mbnl1^–/–^ Mbnl2^+/–^* muscle. Centralized nuclei, a hallmark feature of DM histopathology, are a marker of regenerating myofibers and, in the context of muscular dystrophy, indicate ongoing rounds of muscle degeneration and regeneration. To determine whether the muscles in *Mbnl*-KO mice were experiencing ongoing rounds of regeneration, we also stained the muscles for embryonic myosin heavy chain (eMHC) which denotes regenerating myofibers. Again, we observed the greatest percentage of eMHC^+^ myofibers in the TA and quadriceps muscles followed by small increases in the EDL and diaphragm, while we saw no increase in eMHC^+^ fibers in the soleus or gastrocnemius muscles (Figure 5C). To confirm that the presence of regenerating myofibers was due to muscle degeneration, we also stained the muscles with a high concentration of mouse IgG, which marks necrotic fibers (Supplemental Figure 7, A and B). We again found that the TA muscle had the greatest percentage of necrotic fibers (Supplemental Figure 7, C–H). To determine if the muscles showed any signs of fibrosis or fat accumulation, we stained the muscles with picrosirius red (PR) and perilipin, respectively; however, we noted no evidence of fibrosis or fat accumulation in any muscle examined (data not shown).

### DM1 patient muscles have increased developmental MYH expression and decreased Type II fiber MYH expression.

**Figure 6:**
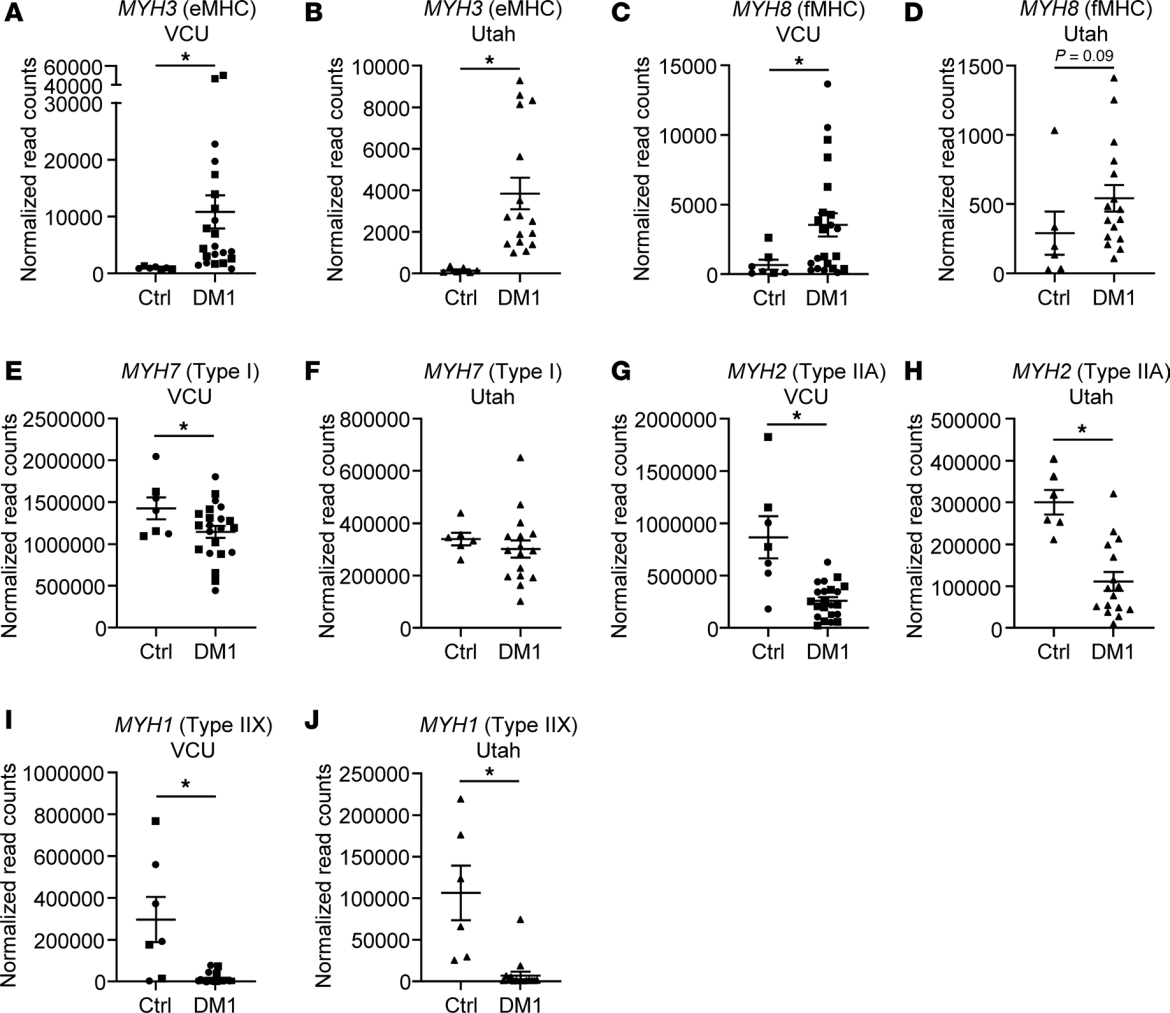
DM1 patient muscles have increased developmental *MYH* expression and decreased Type II fiber *MYH* expression. (**A**–**J**) Normalized read counts of RNA-Seq data from adult healthy controls and adult DM1 muscle biopsies from publicly available datasets for *MYH3* (eMHC) (**A** and **B**), *MYH8* (fMHC) (**C** and **D**), *MYH7* (Type I fibers) (**E** and **F**), *MYH2* (Type IIA fibers) (**G** and **H**), and *MYH1* (Type IIX fibers) (**I** and **J**). VCU dataset unaffected adult controls *n* = 7, adult-onset DM1 *n* = 22. Squares indicate males. Circles indicate females (GSE201255). Utah dataset unaffected adult controls *n* = 6, adult-onset DM1 *n* = 16 (GSE126342) (data are shown as mean ± SEM: *P < 0.05; 1-tailed Student’s *t* test).

Ongoing cycles of degeneration and regeneration are expected in DM1 based on the presence of eMHC^+^ myofibers in DM1 biopsies. More than 0.1% eMHC^+^ fibers is considered abnormal (29), and a study examining 4 patients with DM1 found between 0.3% and 3% eMHC^+^ myofibers in DM1 muscle biopsies (30). To confirm the observation of ongoing rounds of regeneration in a larger cohort of patients with DM1, we pulled RNA-Seq data from 2 publicly available studies comparing adult healthy control and DM1 skeletal muscle biopsies (Gene Expression Omnibus [GEO] accession nos. GSE201255 [Virginia Commonwealth University (VCU)], GSE126342 [Utah]) (31). We confirmed that *MYH3*, which encodes eMHC, is overexpressed in DM1 compared with healthy control muscle (Figure 6, A and B). *MYH8*, encoding fetal MHC (fMHC), is also overexpressed in DM1 muscle (Figure 6, C and D). Both Type I fiber predominance and Type I fiber atrophy have been reported in DM1 (9, 10, 32), so we also examined the expression of the genes encoding Type I (*MYH7*), IIA (*MYH2*), and IIX (*MYH1*) fibers in DM1 patient muscle compared with healthy control muscle. *MYH7* expression is slightly decreased in patients with DM1 in the Virginia Commonwealth University (VCU) dataset (Figure 6E) but not decreased in the Utah dataset (Figure 6F) (GSE201255, GSE126342), most likely reflecting an averaging effect of increased but smaller Type I fibers, as has been reported. In contrast, *MYH2* and *MYH1* expression is massively decreased in DM1 muscle compared with healthy control (Figure 6, G–J), again reflecting the observation of Type II fiber loss in DM1. Unfortunately, no publicly available DM2 RNA-Seq skeletal muscle biopsy datasets exist. These results indicate *Mbnl*-KO muscles recapitulate the muscle degeneration/regeneration occurring in DM1, so the next question we addressed was whether mice also recapitulate the fiber type switching and selective Type I fiber atrophy in DM1.

### Fiber type switching is greater in predominantly fast-twitch glycolytic muscles.

**Figure 7:**
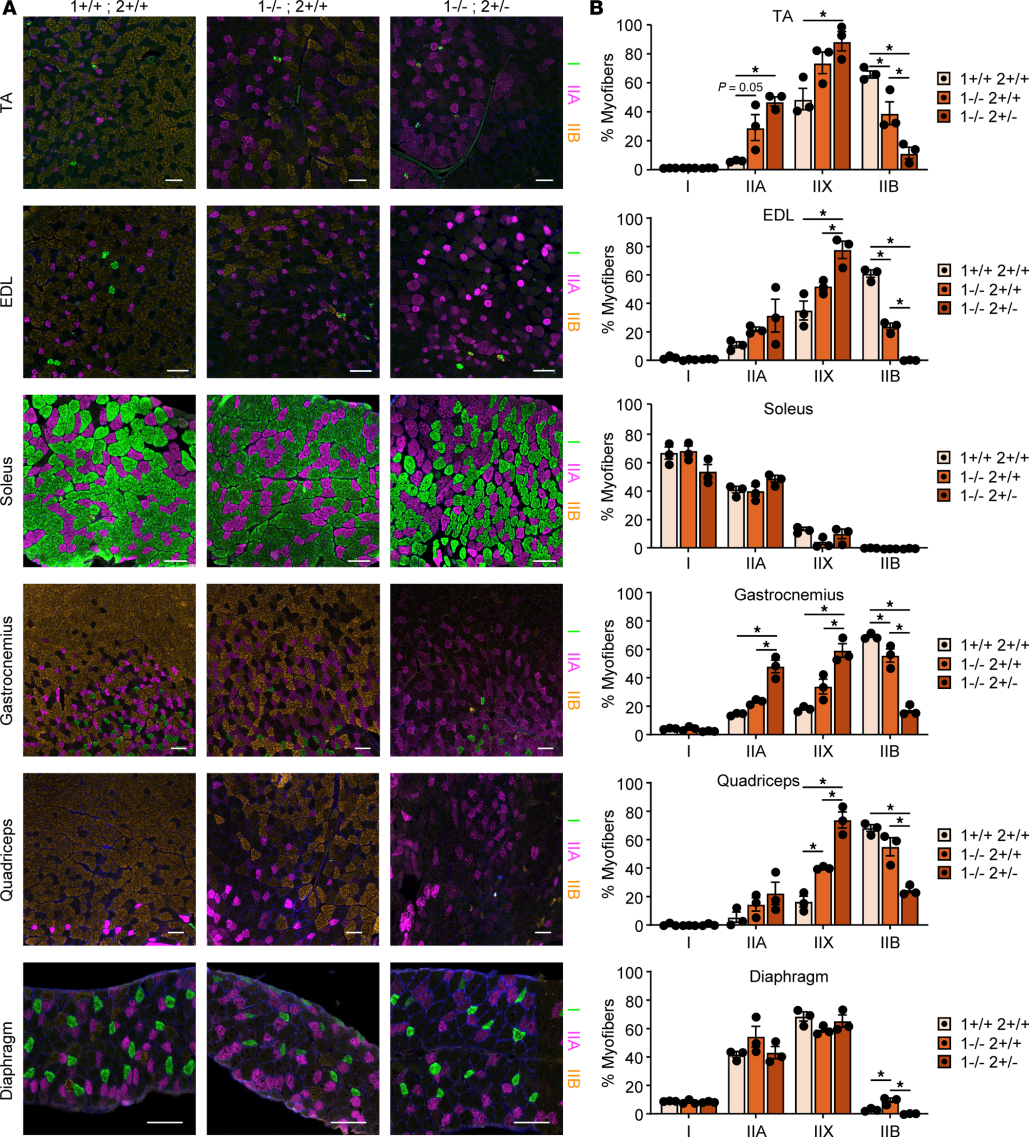
Fiber type switching is greater in predominantly fast-twitch glycolytic muscles. (**A**) Immunofluorescence of Type I (*MYH7*), IIA (*MYH2*), and IIB (*MYH4*) myofibers in muscles from 8-week-old WT, *Mbnl1*^–/–^, and *Mbnl1^–/–^ Mbnl2^+/–^* mice. Scale bar: 100 μM. (**B**) Quantification of percent Type I, IIA, IIX, and IIB fibers per cross-section (data are shown as mean ± SEM, *P < 0.05, 1-way ANOVA with Tukey’s multiple-comparison test). *n* = 3 mice per genotype.

Fiber type switching has previously been reported both in HSA^LR^ and *Mbnl1^–/–^* mice (33). To determine if any mouse muscle displayed greater fiber type switching in response to MBNL loss, we stained each of the muscles for Type I, IIA, IIX, and IIB fibers (listed in order of speed of contraction). Type IIB fibers are a fast-twitch fiber type present in murine muscle that are not present in human muscle. Most muscle fibers express a single MHC, although ~25% of muscle fibers appear hybrid with great variation across muscles and with increased percentages of hybrid fibers with age and disease (34–38). The WT TA muscle is predominantly fast-twitch IIB (~67%) and IIX fibers (~50%) with < 6% type IIA fibers and < 1% Type I fibers, with a small percentage of hybrid fiber types (Figure 7, A and B, and Supplemental Figures 8–10). Note that the presence of hybrid fibers may result in a total greater than 100%. In contrast, the TA muscle from *Mbnl1^–/–^* mice is ~40% IIB, ~75% IIX, and 30% IIA myofibers, a clear shift toward a more oxidative profile, with a loss of the fastest fiber type and a greater percentage of hybrid fibers (Figure 7, A and B, and Supplemental Figures 8–10). This shift becomes even stronger in the TA of *Mbnl1^–/–^ Mbnl2^+/–^* mice with ~12% IIB, ~90% IIX, and ~48% IIA myofibers, an 8-fold increase in IIA fibers, and an almost 2-fold increase in IIX fibers with a corresponding 5.6-fold decrease in IIB fibers (Figure 7, A and B, and Supplemental Figures 8–10). Fiber type changes in the EDL, gastrocnemius, and quadriceps muscles were strikingly similar except for a near-complete loss of IIB fibers in the EDL (Figure 7, A and B, and Supplemental Figure 8). Greater changes in fiber type were observed in the bone proximal regions of the TA, gastrocnemius, and quadriceps muscles (Supplemental Figures 8 and 9). In the gastrocnemius, the lateralis appears to have greater fiber type shifting compared with the medialis (Supplemental Figures 8 and 9). In contrast, there was virtually no fiber type switching in the already highly oxidative soleus and diaphragm muscles (Figure 7, A and B, and Supplemental Figures 8–10).

### The fiber atrophy/hypertrophy profile in Mbnl-KO mice looks more like DM1 than DM2.

Because there is selective atrophy of Type I myofibers and hypertrophy of Type II myofibers in DM1 and selective atrophy of Type II myofibers in DM2 (9, 10), we performed myofiber size distribution analysis for each fiber type across the muscle set to determine if there was atrophy of any fiber type following MBNL loss or if each fiber type displayed equivalent hypertrophy. There are very few Type I fibers in the TA, comprising < 0.6% of the myofibers, and there is no overt difference in the size of these fibers with loss of MBNL (Supplemental Figure 11A). This is in contrast with the EDL muscle, which — while it also only has 0.8%–2.4% Type I fibers — has a clear atrophy of these fibers (Figure 7 and Supplemental Figure 11B). The soleus, composed of > 50% Type I fibers, shows only a slight atrophy of these fibers (Supplemental Figure 11C). There is no obvious atrophy of Type I fibers in the gastrocnemius (Supplemental Figure 11D), but there is clear atrophy of Type I fibers in the diaphragm (Supplemental Figure 11E). There is a striking shift toward hypertrophy of Type IIA fibers in the TA muscle of both *Mbnl1^–/–^* and *Mbnl1^–/–^ Mbnl2^+/–^* mice (Supplemental Figure 12A), while there is only an increase in the Type IIA myofiber size variability in the EDL of *Mbnl1^–/–^ Mbnl2^+/–^* mice (Supplemental Figure 12B). There is no obvious change in Type IIA myofiber size in the soleus with MBNL loss (Supplemental Figure 12C), but there is a modest shift toward hypertrophy of IIA fibers in the gastrocnemius that is progressive with the amount of MBNL loss (Supplemental Figure 12D). In contrast, there is only a slight hypertrophy of IIA fibers in the quadriceps of *Mbnl1^–/–^* mice that disappears in *Mbnl1^–/–^ Mbnl2^+/–^* mice (Supplemental Figure 12E). There is no change in the size of Type IIA fibers in the diaphragm (Supplemental Figure 12F). The fiber size distribution of IIX fibers largely mirrors that of IIA, with clear hypertrophy in the TA, minimal change in the EDL and soleus, an equivalent shift in hypertrophy of IIX fibers in *Mbnl1^–/–^* and *Mbnl1^–/–^ Mbnl2^+/–^* gastrocnemius, slight hypertrophy in quadriceps that is greater in *Mbnl1^–/–^* mice than *Mbnl1^–/–^ Mbnl2^+/–^* mice, and hypertrophy of IIX fibers in the diaphragm only in *Mbnl1^–/–^ Mbnl2^+/–^* mice (Supplemental Figure 13, A–F). The TA, EDL, gastrocnemius, and quadriceps muscles all display loss of Type IIB fibers (Figure 7, A and B, and Supplemental Figure 8), but the remaining IIB fibers differ in size between the muscles (Supplemental Figure 14, A–F). In the TA and quadriceps, IIB fibers of *Mbnl1^–/–^* mice display hypertrophy, while those of *Mbnl1^–/–^ Mbnl2^+/–^* mice display atrophy (Supplemental Figure 14, A and E). In the EDL and diaphragm, there is no change in the size of IIB fibers in *Mbnl1^–/–^* mice but atrophy in *Mbnl1^–/–^ Mbnl2^+/–^* mice (Supplemental Figure 14, B and F). This atrophy is much clearer in the EDL than the diaphragm (Supplemental Figure 14, B and F). Meanwhile, there is no change in IIB fiber size in the soleus or gastrocnemius in either *Mbnl1^–/–^* or *Mbnl1^–/–^ Mbnl2^+/–^* mice (Supplemental Figure 14, C and D). To summarize, glycolytic muscles such as the TA, EDL, gastrocnemius, and quadriceps display much greater fiber type switching than oxidative muscles such as the soleus and diaphragm, and the fiber atrophy/hypertrophy profile across muscles resembles a more DM1- than DM2-like appearance with atrophy of Type I myofibers in some muscles and hypertrophy of Type IIA and IIX fibers in most muscles.

### Degree of missplicing does not explain differential muscle susceptibility.

**Figure 8:**
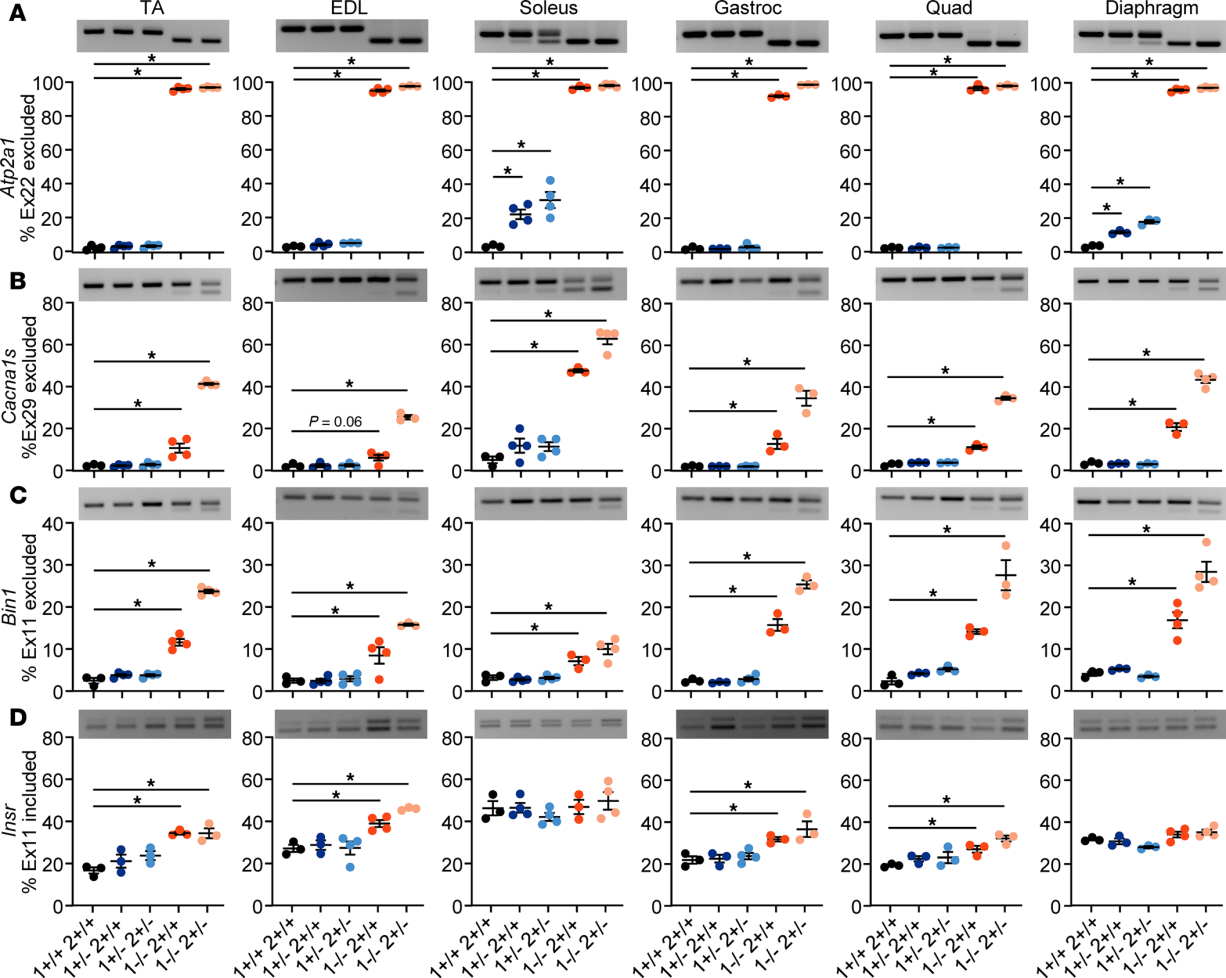
Degree of missplicing does not explain differential muscle susceptibility. (**A**–**D**) RT-PCR of *Atp2a1* exon 22 (**A**), *Cacna1s* exon 29 (**B**), *Bin1* exon 11 (**C**), and *Insr* exon 11 (**D**) alternative splicing in 8wk old mouse muscles with genetic titration of MBNL (data are shown as mean ± SEM, *P < 0.05, 1-way ANOVA with Dunnett’s multiple-comparison test). *n* = 3–4 mice per genotype.

The MBNL proteins are RBPs that function in the regulation of RNA alternative splicing and polyadenylation in the nucleus and RNA localization in the cytoplasm (39–42). The resultant spliceopathy from MBNL sequestration is well characterized in DM muscle, and there is a correlation between missplicing and muscle disease severity as determined by ankle dorsiflexion weakness (13, 15, 43), quantitative myometry (13), handgrip strength (13, 15, 43), and 10-meter walk/running speed (15, 43). We therefore decided to test if mouse muscles with greater DM-relevant histopathology following MBNL loss have a greater degree of missplicing. Considerable effort has been put forth to identify a composite measure of differentially spliced events that reflect the range of phenotypic severity observed in DM (13, 15, 44, 45). The most recent composite RNA splicing metric utilized in patients with DM1, the Myotonic Dystrophy Splice Index (SI), detects 22 splicing events (15). The SI normalizes the relative degree of splicing dysregulation and correlates with multiple measures of muscle performance. We therefore examined a subset of these known MBNL-regulated splicing events, which also occur in mice and that have been independently shown to correlate with muscle weakness: *Atp2a1* exon 22, *Bin1* exon 11, *Cacna1s* exon 29, *Clcn1* exon 7a, *Dmd* exon 78, *Mbnl1* exon 5, *Nfix* exon 8, and *Insr* exon 11 (13, 15). *Dmd* exon 78 exclusion was not reliably detected across muscles, so these data are not shown. For 3 of the splicing events examined (*Clcn1*, *Mbnl1*, and *Nfix*), while we observed clear missplicing following genetic titration of MBNL, we observed little difference in the degree of missplicing across muscles (Supplemental Figure 15, A–C). In contrast, we were surprised to find missplicing of *Atp2a1* with less genetic titration of MBNL only in the soleus and diaphragm muscles, requiring only heterozygosity of *Mbnl1* (*Mbnl1^+/–^*) to see exclusion of exon 22 (Figure 8A). This was more severe in the soleus than the diaphragm. All muscles showed a similar level of *Atp2a1* missplicing with *Mbnl1^–/–^* or *Mbnl1^–/–^ Mbnl2^+/–^*. We observed a similar result for *Cacna1s* exon 29 exclusion, where we saw a greater degree of *Cacna1s* missplicing in the soleus of all genotypes compared with other muscles (Figure 8B). Conversely, we observed less missplicing of *Bin1* in the soleus of *Mbnl1^–/–^* and *Mbnl1^–/–^ Mbnl2^+/–^* mice (Figure 8C), and we observed no missplicing of *Insr* in the soleus or diaphragm of any genotype compared with modest missplicing of *Insr* in the TA, EDL, gastrocnemius, and quadriceps of *Mbnl1^–/–^* and *Mbnl1^–/–^ Mbnl2^+/–^* mice (Figure 8D). In conclusion, the degree of missplicing across the muscles failed to correlate with the differences observed histologically.

## Discussion

Many hypotheses have been proposed to explain the muscle involvement patterns across the muscular dystrophies, including differences in contractile function, force, and load bearing across muscles, differences in the types of proteins mutated such as membrane-bound versus sarcomeric (46, 47), and differential expression of muscle genes and isoforms (48). However, most of these hypotheses are less likely to explain the muscle involvement patterns in DM1 and DM2, particularly since the mutations in DM are not in membrane-bound or sarcomeric proteins typical of many other muscular dystrophies, and rather than loss-of-function mutations, the DMs are caused by gain-of-function mutations. Nevertheless, there could certainly be a role for the differential expression of muscle genes and isoforms.

Despite a shared pathomechanism of MBNL sequestration in DM1 and DM2, the muscle involvement patterns are different, indicating differential muscle susceptibility to pathogenic repeat expansion mutations both within individuals and between DM1 and DM2. One hypothesis for this differential susceptibility is that the expression of the host genes (*DMPK*, *CNBP*) differs among muscles, leading to a higher pathogenic repeat load in more severely affected muscles. We tested this by analyzing RNA-Seq data from a healthy volunteer transcriptome atlas of 6 leg muscles. While we did not detect a large variation in *DMPK* expression across the muscles examined, there is greater variability in the expression of *MBNL1*, leading to *DMPK/MBNL1* ratios that loosely correlate with the degree of fat infiltration in these muscles in DM1 determined by MRI in other studies (7). One caveat to this result is that there was only 1 distal muscle, the gastrocnemius lateralis, examined, and distal muscles are more affected in DM1, with the gastrocnemius medialis, soleus, flexor hallucis longus, and TA showing the greatest degree of fat infiltration (7). Expression data are needed from additional highly affected distal muscles to reach more definitive conclusions. In DM2, proximal muscles tend to have higher fat accumulation by MRI with less distal involvement (4, 5, 8). *CNBP* expression is lower in the typically less-affected distal gastrocnemius muscle than the more-affected proximal muscles, correlating with muscle involvement. However, when the ratio of *CNBP*/*MBNL1* is examined, this correlation is lost, perhaps because of the involvement of additional RBPs such as RBFOX1/2 in DM2 pathogenesis (16), although *RBFOX1* and *RBFOX2* have similar expression profiles to *MBNL1* across muscles, perhaps indicating yet unknown mechanisms of susceptibility.

One constraint of our study is that we only examined the expression of genes involved in the disease pathway in healthy human muscle, which may not reflect the expression patterns of diseased muscle. Previous DM1 studies have identified impaired isolation of transcripts containing CUG expansions using traditional RNA extraction methodologies, resulting in lower-than-accurate transcript level measurements, possibly due to transcript retention in RNA foci (21, 49). The use of healthy samples alleviates this concern; however, the presence of repeat expansions and subsequent loss of MBNL proteins is known to alter the gene expression program of cells in which they are expressed (41, 50–52). It remains unclear, therefore, whether *DMPK* and *CNBP* expression patterns remain consistent once they contain repeat expansions. Further studies are needed to examine *DMPK*, *CNBP*, and *MBNL* expression levels across affected and unaffected muscles in patients with DM, with careful attention paid to RNA extraction methodology. The presence of repeat expansions in both *DMPK* and *CNBP* have been shown to decrease expression of the proteins encoded by these host genes (53–55), and haploinsufficiency of these genes has been suggested as possibly contributing to disease. This is less likely in DM1, as KO of *Dmpk* in mice has shown no effect on muscle histology, strength, or myotonia (56). KO or heterozygosity of *Cnbp* (formerly *Znf9*), however, does have an effect on muscle with increased centralized nuclei, disrupted sarcomeric structure, and myotonic discharges (54, 57, 58). It may therefore be possible that haploinsufficiency of *CNBP* plays a larger role in DM2 muscle pathology than *DMPK* haploinsufficiency in DM1. It may be interesting to explore heterozygosity of *Cnbp* in addition to *Mbnl* loss in future studies to determine if this promotes a more DM2-like histopathology in mouse muscle.

Regardless of the possible contributions of host gene haploinsufficiency, the primary mechanism of pathogenicity in DM1 and DM2 has long been understood to be that of RNA-mediated toxicity, whereby the pathogenic repeat–containing RNAs sequester the MBNL proteins (59–61). We therefore sought to determine whether mice recapitulate this differential muscle susceptibility, to identify the ideal mouse muscles for outcome measure testing, and to determine which aspects of DM muscle pathology are contributed by loss of MBNL function. We found that mouse muscles are differentially affected by loss of MBNL, as the TA muscle develops many more histological signs of disease than any of the other muscles examined, followed by the quadriceps. The mouse soleus muscle, in contrast, is relatively spared, with no increase in centralized nuclei, eMHC^+^ fibers, necrotic fibers, or fiber type switching. This pattern of involvement does not quite resemble DM1 or DM2, and *Mbnl*-KO mice do not recapitulate the distal-proximal or proximal-distal gradients observed in either disease, respectively. Instead, the 2 most affected mouse muscles examined are 1 distal and 1 proximal muscle. This may be due to intrinsic differences between human and mouse muscles or differences between bipedal and quadrupedal locomotion. One notable difference between human and mouse muscles is the presence of an additional fast twitch MHC (*MYH4*) in mouse muscles, creating the fiber type IIB. We observe greater pathology in the more glycolytic muscles (TA, EDL, quadriceps, gastrocnemius) with relatively spared pathology in the more oxidative muscles (soleus, diaphragm). These findings should translate to new mouse models of both DM1 and DM2 that have repeats inserted into their endogenous loci as the TA and quadriceps muscles have the highest ratio of *Dmpk*/MBNL1 and *Cnbp*/MBNL1.

Fiber typing of mouse muscles following loss of MBNL resembles a more DM1-like pathology than DM2, as there is a shift toward a more oxidative profile, mirroring the predominance of Type I myofibers in DM1 (9, 10). Like DM1, we also observe atrophy of Type I myofibers in the mouse EDL and diaphragm muscles with very slight atrophy in the soleus. Type II fiber hypertrophy has also been reported in DM1, which was also observed in the Type IIA and IIX fibers of *Mbnl*-KO muscles. In contrast, Type II fiber atrophy was reported in DM2, which we did not observe except for type IIB fibers (not present in humans) in the muscles of *Mbnl1^–/–^ Mbnl2^+/–^* mice.

Given that loss or sequestration of MBNL proteins leads to broad spliceopathy (26, 41), we asked if the degree of missplicing across the muscles could explain the differences in histological severity. We were surprised to find similar levels of missplicing across most events among the muscles following genetic titration of MBNL, indicating that greater missplicing did not explain the differences in how the muscles were affected. For some events, such as *Atp2a1* and *Cacna1s*, we even observed earlier or more severe missplicing in the soleus muscle where we did not see histopathology. One explanation for this, at least for *Atp2a1*, could be that the levels of *ATP2A1* are lower in Type I than Type II fibers (62), and the soleus has the highest percentage of Type I fibers. This lower expression of *Atp2a1* in the soleus could lead to missplicing with less MBNL titration. There is not a significant difference in the expression of *CACNA1S* across fiber types, however, indicating that this explanation does not account for this splicing event. An additional explanation for this observation would be differential expression of additional RBPs across muscles that coregulate many MBNL regulated splicing events, although Western blotting of CELF1, RBFOX1, and HNRNPA1 did not detect significantly higher levels of any of these RBPs in the soleus compared with any other muscle. We tested almost half of the MBNL-regulated splicing events that occur in both humans and mice and that are known to correlate with muscle weakness (13), and we did not find significantly greater missplicing in the more histologically affected muscles. A future transcriptome-wide analysis could be used to confirm this finding, although the more interesting analysis may lie in gene expression or alternative polyadenylation changes across the muscles, as MBNL plays important roles in these processes as well.

The observation of similar levels of *Clcn1* missplicing across the muscles raises interesting questions about myotonia. Previous work has shown that HSA^LR^ soleus and diaphragm muscles do not develop myotonia, while the EDL does, presumably with similar levels of missplicing (20). Patients with congenital DM1 (CDM) also have a striking lack of myotonia in childhood that develops later in life, despite early missplicing of *CLCN1* (43). While it’s clear that missplicing or loss of function of *Clcn1* directly causes myotonia (63–66), there appear to be molecular modifiers that allow certain muscles to avoid myotonia and children with CDM to avoid the early onset of the symptom. Similarly, it has been hypothesized that missplicing of *Clcn1* leads to the fiber type transition in TA muscles of LR41; *Mbn1*^–/–^ mice as correction of *Clcn1* missplicing reverses the muscle fiber type transition (33); however, we observe equivalent levels of *Clcn1* missplicing in muscles that do not develop fiber type switching, indicating further that either molecular modifiers in some muscles prevent this phenomenon from occurring or rather more likely induce this fiber type pattern even in the setting of normal *Clcn1* splicing.

In summary, while the ratio of *DMPK/MBNL1* and the levels of *CNBP* reflect muscle susceptibility to some degree in DM1 and DM2, our evidence of MBNL loss alone differentially affecting mouse muscles suggests that it is more complicated than this and highlights a possible role for yet-to-be-identified modifier genes. While MBNL-dependent misregulated splicing does not appear to be the cause of the differential muscle pathology, MBNL regulates many other cellular processes, including gene expression, alternative polyadenylation, and RNA localization, which may contribute to existing intrinsic differences across the muscles and impact how they are affected. These findings point to a role for MBNL loss in muscle pattern involvement in DM1, indicate additional mechanisms of pathogenicity beyond MBNL sequestration in DM2, and have important implications for the muscle of choice (such as selecting the TA muscle for histological analysis or the soleus muscle for splicing analysis) when performing analyses in mouse models and evaluating potentially new therapeutic modalities and biomarkers.

## Methods

### Sex as a biological variable.

Male and female mice were used for our studies; however, quantification of histological and immunofluorescence data is shown only for male mice, as muscle data from male and female mice cannot be combined due to muscle weight and fiber size variance between the sexes (67). Similar results were observed in female mice.

### Mice.

The generation of *Mbnl1*^ΔE3/ΔE3^ (*Mbnl1*^–/–^) and *Mbnl2*^+/ΔE2^ (*Mbnl2*^+/–^) mice has been previously described (26, 68, 69). Compound KOs were generated by crossing a *Mbnl1*^+/–^ *Mbnl2*^+/+^ dam (congenic FVB/NJ) to a *Mbnl1*^+/–^ *Mbnl2*^+/–^ stud (congenic FVB/NJ). Unless otherwise specified, muscles were collected from all mice at 8 weeks of age, the time point at which *Mbnl1*^–/–^ *Mbnl2*^+/–^ (FVB/NJ) reach terminal endpoint. WT FVB/NJ mice used in Figure 2 and Supplemental Figure 2 were obtained from The Jackson Laboratory. All mice were housed at the University of Florida.

### Histology.

Muscles were embedded in OCT, frozen in liquid nitrogen–cooled isopentane, and stored at –80°C until further analysis. Sections (10 μm thick) were stained for hematoxylin (Polysciences, 24244) and eosin (Polysciences, 09859) (H&E) or PR. For PR staining, slides were fixed in 4% PFA for 45 minutes before following standard protocols. PR solution contained 0.1% Direct Red 80 in saturated Picric acid. Slides were scanned at 20× using a Motic Slide scanner and .tif files exported using Leica Aperio ImageScope software.

### Immunofluorescence.

Slides were blocked for 45 minutes in 5% donkey serum and 0.3% TritonX-100 in PBS. For mouse primary antibodies, donkey anti-mouse Fab fragments (Jackson ImmunoResearch, 715-007-003) were added to the blocking buffer (1:50). Primary antibodies were incubated for 3 hours at room temperature in blocking solution, followed by 3 washes with PBS-T, and 1-hour incubations in secondary antibody. Slides were mounted with FluorSave. Antibodies included the following: Laminin (MilliporeSigma, L9393, 1:1,000), eMHC/*MYH3* (Developmental Studies Hybridoma Bank [DSHB], F1.652-s, 1:40), MHC-I (DSHB, BA-F8-c, 1:400), MHC-IIA (DSHB, SC-71-c, 1:300), MHC-IIB (DSHB, BF-F3-c, 1:200), MHC-IIX (DSHB, 6H1-s, 1:20), DAPI (MilliporeSigma, D9542, 1:25,000), and Perilipin (Cell Signaling Technology, 9349S, 1:1000). For staining necrotic fibers, α–mouse IgG-488 was used at 1:100. Secondary antibodies used include anti–mouse IgG-488 (Thermo Fisher Scientific, A11029), anti–rabbit IgG-568 (Thermo Fisher Scientific, A11036), anti–rabbit-405 (Thermo Fisher Scientific, A48254), anti–mouse IgG1-568 (Thermo Fisher Scientific, sms1AF568-1), anti–mouse IgG2b-488 (Thermo Fisher Scientific, SA5-10371-AFP), and anti-mouse IgM-647 (Jackson ImmunoResearch, 715-605-020). Images were acquired with an Echo Revolution automated microscope.

### Image quantification.

For total myofiber number quantification and myofiber size distribution analysis, whole-muscle cross-section images stained for Laminin were segmented by Cellpose (70) using the modified GoogleColab script written by Ariel Waisman followed by myofiber identification using the FIJI plugin LabelstoROIs (71). Misidentified myofibers were manually corrected. Centralized nuclei were counted manually for 1 whole cross-section per muscle and were represented as a percentage of the total myofibers per muscle cross-section. eMHC^+^ fibers were counted manually for 2 cross-sections per muscle and were then averaged and represented as a percentage of the total myofibers per muscle cross-section. Fiber type was determined by thresholding mean pixel intensity of each channel using the ROIs obtained from LabelstoROIs in FIJI. Diaphragm thickness was measured at 3 locations along the length of the muscle for 3 cross-sections per muscle and averaged.

### RNA isolation, cDNA synthesis, and quantitative PCR (qPCR).

RNA from tissues was isolated using NucleoZOL and NucleoSpin RNA Set for NucleoZOL (Macherey-Nagel) according to the manufacturer’s protocol. In total, 1 μg of input RNA was used for cDNA synthesis with random hexamers using the Promega GoScript Reverse Transcription System, followed by an RNase H treatment to remove the RNA template. qPCR was performed using the RT² SYBR Green qPCR Mastermix (Qiagen), according to manufacturer’s instructions. Relative expression levels were calculated by normalizing to the geometric mean of 3 housekeeping genes (*Atp6v1c1*, *Eif3g*, *Eapp*) identified by Hicks et al. (19). Assays were performed in triplicate. Primer sequences are listed in Supplemental Table 1.

### Splicing analysis.

Alternative splicing products were generated following reverse transcription PCR (RT-PCR) using GoTaq G2 Green MasterMix (Promega), separated on agarose gels, and visualized with the Bio-Rad Molecular Imager ChemiDoc XRS+. Signal intensities were calculated using Bio-Rad Image Lab Software. Primer sequences are listed in Supplemental Table 1.

### Analysis of RNA-Seq data.

Human DM1 and healthy control muscle RNA-Seq data (FASTQ files) were retrieved from the GEO database (GSE201255, GSE126342). Healthy human muscle RNA-Seq data (FASTQ files) were obtained from the European Genome-phenome Archive (EGA) (EGAD00001008657). FASTQ files were processed with Salmon (72). The R package tximport was used to prepare gene level count data from salmon output files (73). Normalized count data were then derived using DESeq2 (74).

### Western blotting.

Muscles were lysed in protein lysis buffer (50 mM Tris-HCl [pH 7.5], 150 mM NaCl, 5 mM EDTA, 1% Igepal, 0.25% sodium deoxycholate, and protease and phosphatase inhibitors) followed by Bead-Ruptor homogenization. Protein lysates (40 μg) were separated on AnykD TGX Stain-Free Mini-PROTEAN or CRITERION gels (Bio-Rad), imaged in a ChemiDoc XRS+ system and transferred to a PVDF membrane. Blotting was performed using antibodies against MBNL1 (1:1,000, A2764, gift from Charles Thornton, University of Rochester, Rochester, New York, USA), RBFOX1 (1:250, gift from Thomas Cooper, Baylor College of Medicine, Houston, Texas, USA), CELF1 (1:1,000, 3B1, Santa Cruz Biotechnology Inc.), and HNRNPA1/A1B (1:1,000, 9H10, gift from Gideon Dreyfuss, University of Pennsylvania, Philadelphia, Pennsylvania, USA). HRP-conjugated anti-mouse and anti-rabbit secondaries were used at 1:10,000–1:20,000. Signal intensity was calculated using Bio-Rad Image Lab Software. Blots were normalized to total protein detected using Bio-Rad’s stain-free imaging technology.

### Statistics.

Statistical significance was determined in GraphPad Prism by ordinary 1-way ANOVA with Tukey’s multiple-comparison test or 1-tailed Student’s *t* test, unless otherwise specified, and all statistical analyses were based on at least 3 biologically independent samples.

### Study approval.

All animal procedures were conducted in accordance with NIH guidelines and approved by the IACUC at the University of Florida (protocol nos. 20203677 and 20230652).

### Data availability.

Human DM1 datasets were obtained from the publicly available GEO database (GSE201255, GSE126342). Healthy human muscle controlled data were accessed through the Data Access Committee (EGAC00001002603) of the European Genome-phenome Archive (EGA), study no. EGAS00001005904 (dataset EGAD00001008657). Values for all data points in graphs are reported in the Supporting Data Values file.

## Author contributions

MLD designed the project; MLD, AF, GMV, MFADM, and JLB generated and analyzed data; MLD wrote the manuscript; MSS provided funding and research support. All authors read and approved the final manuscript.

## Supplementary Material

Supplemental data

Unedited blot and gel images

Supplemental table 1

Supporting data values

## Version 1 08/14/2025

In-Press Preview

## Version 2 09/23/2025

Electronic publication

## References

[1] Bouchard C, Tremblay JP (2023). Limb-girdle muscular dystrophies classification and therapies. J Clin Med.

[2] Tihaya MS (2023). Facioscapulohumeral muscular dystrophy: the road to targeted therapies. Nat Rev Neurol.

[3] Wenninger S (2018). Core clinical phenotypes in myotonic dystrophies. Front Neurol.

[4] Peric S (2017). Magnetic resonance imaging of leg muscles in patients with myotonic dystrophies. J Neurol.

[5] Kornblum C (2006). Distinct neuromuscular phenotypes in myotonic dystrophy types 1 and 2: a whole body highfield MRI study. J Neurol.

[6] Heskamp L (2019). Lower extremity muscle pathology in myotonic dystrophy type 1 assessed by quantitative MRI. Neurology.

[7] Garibaldi M (2022). Muscle magnetic resonance imaging in myotonic dystrophy type 1 (DM1): refining muscle involvement and implications for clinical trials. Eur J Neurol.

[8] Madrid DA (2023). Associations between lower extremity muscle fat fraction and motor performance in myotonic dystrophy type 2: a pilot study. Muscle Nerve.

[9] Meola G, Cardani R (2015). Myotonic dystrophies: an update on clinical aspects, genetic, pathology, and molecular pathomechanisms. Biochim Biophys Acta.

[10] Nadaj-Pakleza A (2011). Muscle pathology in myotonic dystrophy: light and electron microscopic investigation in eighteen patients. Folia Morphol (Warsz).

[11] Bassez G (2008). Type 2 myotonic dystrophy can be predicted by the combination of type 2 muscle fiber central nucleation and scattered atrophy. J Neuropathol Exp Neurol.

[12] Pisani V (2008). Preferential central nucleation of type 2 myofibers is an invariable feature of myotonic dystrophy type 2. Muscle Nerve.

[13] Nakamori M (2013). Splicing biomarkers of disease severity in myotonic dystrophy. Ann Neurol.

[14] Otero BA (2021). Transcriptome alterations in myotonic dystrophy frontal cortex. Cell Rep.

[15] Provenzano M (2025). The Splice Index as a prognostic biomarker of strength and function in myotonic dystrophy type 1. J Clin Invest.

[16] Sellier C (2018). rbFOX1/MBNL1 competition for CCUG RNA repeats binding contributes to myotonic dystrophy type 1/type 2 differences. Nat Commun.

[17] Abbassi-Daloii T (2023). A transcriptome atlas of leg muscles from healthy human volunteers reveals molecular and cellular signatures associated with muscle location. Elife.

[18] Mankodi A (2000). Myotonic dystrophy in transgenic mice expressing an expanded CUG repeat. Science.

[19] Hicks SM (2024). Alternative splicing dysregulation across tissue and therapeutic approaches in a mouse model of myotonic dystrophy type 1. Mol Ther Nucleic Acids.

[20] Moyer M (2011). Differential susceptibility of muscles to myotonia and force impairment in a mouse model of myotonic dystrophy. Muscle Nerve.

[21] Nutter CA (2019). Cell-type-specific dysregulation of RNA alternative splicing in short tandem repeat mouse knockin models of myotonic dystrophy. Genes Dev.

[22] Kuyumcu-Martinez NM (2007). Increased steady-state levels of CUGBP1 in myotonic dystrophy 1 are due to PKC-mediated hyperphosphorylation. Mol Cell.

[23] Cardani R (2013). Overexpression of CUGBP1 in skeletal muscle from adult classic myotonic dystrophy type 1 but not from myotonic dystrophy type 2. PLoS One.

[24] Ward AJ (2010). CUGBP1 overexpression in mouse skeletal muscle reproduces features of myotonic dystrophy type 1. Hum Mol Genet.

[25] Li M (2020). HNRNPA1-induced spliceopathy in a transgenic mouse model of myotonic dystrophy. Proc Natl Acad Sci U S A.

[26] Lee K-Y (2013). Compound loss of muscleblind-like function in myotonic dystrophy. EMBO Mol Med.

[27] Ivanovic V (2023). Clinical score for early diagnosis of myotonic dystrophy type 2. Neurol Sci.

[29] Sewry CA (2021). Importance of immunohistochemical evaluation of developmentally regulated myosin heavy chains in human muscle biopsies. Neuromuscul Disord.

[30] Thornell LE (2009). Satellite cell dysfunction contributes to the progressive muscle atrophy in myotonic dystrophy type 1. Neuropathol Appl Neurobiol.

[31] Hale MA (2023). Dynamics and variability of transcriptomic dysregulation in congenital myotonic dystrophy during pediatric development. Hum Mol Genet.

[32] Tohgi H (1994). Muscle histopathology in myotonic dystrophy in relation to age and muscular weakness. Muscle Nerve.

[33] Hu N (2023). Correction of Clcn1 alternative splicing reverses muscle fiber type transition in mice with myotonic dystrophy. Nat Commun.

[34] Medler S (2019). Mixing it up: the biological significance of hybrid skeletal muscle fibers. J Exp Biol.

[35] Klitgaard H (1990). Ageing alters the myosin heavy chain composition of single fibres from human skeletal muscle. Acta Physiol Scand.

[36] St-Jean-Pelletier F (2017). The impact of ageing, physical activity, and pre-frailty on skeletal muscle phenotype, mitochondrial content, and intramyocellular lipids in men. J Cachexia Sarcopenia Muscle.

[37] Andersen JL (1999). Increase in the degree of coexpression of myosin heavy chain isoforms in skeletal muscle fibers of the very old. Muscle Nerve.

[38] Ciciliot S (2013). Muscle type and fiber type specificity in muscle wasting. Int J Biochem Cell Biol.

[39] Ho TH (2004). Muscleblind proteins regulate alternative splicing. EMBO J.

[40] Pascual M (2006). The Muscleblind family of proteins: an emerging class of regulators of developmentally programmed alternative splicing. Differentiation.

[41] Wang ET (2012). Transcriptome-wide regulation of pre-mRNA splicing and mRNA localization by muscleblind proteins. Cell.

[42] Batra R (2014). Loss of MBNL leads to disruption of developmentally regulated alternative polyadenylation in RNA-mediated disease. Mol Cell.

[43] Hartman JM (2024). RNA mis-splicing in children with congenital myotonic dystrophy is associated with physical function. Ann Clin Transl Neurol.

[44] Tanner MK (2021). Targeted splice sequencing reveals RNA toxicity and therapeutic response in myotonic dystrophy. Nucleic Acids Res.

[45] Wagner SD (2016). Dose-dependent regulation of alternative splicing by MBNL proteins reveals biomarkers for myotonic dystrophy. PLoS Genet.

[46] Khlebtovsky A (2018). A hypothesis for mechanisms of weakness distribution in muscular dystrophies. J Neurol Disord.

[47] Ozisik O (2024). System-level analysis of genes mutated in muscular dystrophies reveals a functional pattern associated with muscle weakness distribution. Sci Rep.

[48] Huovinen S (2015). Differential isoform expression and selective muscle involvement in muscular dystrophies. Am J Pathol.

[49] Davis BM (1997). Expansion of a CUG trinucleotide repeat in the 3’ untranslated region of myotonic dystrophy protein kinase transcripts results in nuclear retention of transcripts. Proc Natl Acad Sci U S A.

[50] Botta A (2007). Gene expression analysis in myotonic dystrophy: indications for a common molecular pathogenic pathway in DM1 and DM2. Gene Expr.

[51] Osborne RJ (2009). Transcriptional and post-transcriptional impact of toxic RNA in myotonic dystrophy. Hum Mol Genet.

[52] Todd PK (2014). Transcriptional changes and developmental abnormalities in a zebrafish model of myotonic dystrophy type 1. Dis Model Mech.

[53] Furling D (2003). Changes in myotonic dystrophy protein kinase levels and muscle development in congenital myotonic dystrophy. Am J Pathol.

[54] Wei C (2018). Reduction of cellular nucleic acid binding protein encoded by a myotonic dystrophy type 2 gene causes muscle atrophy. Mol Cell Biol.

[55] Raheem O (2010). Mutant (CCTG)n expansion causes abnormal expression of zinc finger protein 9 (ZNF9) in myotonic dystrophy type 2. Am J Pathol.

[56] Carrell ST (2016). Dmpk gene deletion or antisense knockdown does not compromise cardiac or skeletal muscle function in mice. Hum Mol Genet.

[57] Chen W (2007). Haploinsuffciency for Znf9 in Znf9+/- mice is associated with multiorgan abnormalities resembling myotonic dystrophy. J Mol Biol.

[58] Coni S (2021). Translational control of polyamine metabolism by CNBP is required for Drosophila locomotor function. Elife.

[59] Miller JW (2000). Recruitment of human muscleblind proteins to (CUG) (n) expansions associated with myotonic dystrophy. EMBO J.

[60] Warf MB, Berglund JA (2007). MBNL binds similar RNA structures in the CUG repeats of myotonic dystrophy and its pre-mRNA substrate cardiac troponin T. RNA.

[61] Mankodi A (2001). Muscleblind localizes to nuclear foci of aberrant RNA in myotonic dystrophy types 1 and 2. Hum Mol Genet.

[62] Murgia M (2021). Protein profile of fiber types in human skeletal muscle: a single-fiber proteomics study. Skelet Muscle.

[63] Mankodi A (2002). Expanded CUG repeats trigger aberrant splicing of ClC-1 chloride channel pre-mRNA and hyperexcitability of skeletal muscle in myotonic dystrophy. Mol Cell.

[64] Lueck JD (2007). Chloride channelopathy in myotonic dystrophy resulting from loss of posttranscriptional regulation for CLCN1. Am J Physiol Cell Physiol.

[65] Steinmeyer K (1991). Inactivation of muscle chloride channel by transposon insertion in myotonic mice. Nature.

[66] Gurnett CA (1995). Absence of the skeletal muscle sarcolemma chloride channel ClC-1 in myotonic mice. J Biol Chem.

[67] Davenport ML (2024). Spiny mice are primed but fail to regenerate volumetric skeletal muscle loss injuries. Skelet Muscle.

[68] Kanadia RN (2003). A muscleblind knockout model for myotonic dystrophy. Science.

[69] Charizanis K (2012). Muscleblind-like 2-mediated alternative splicing in the developing brain and dysregulation in myotonic dystrophy. Neuron.

[70] Stringer C (2021). Cellpose: a generalist algorithm for cellular segmentation. Nat Methods.

[71] Waisman A (2021). Automatic and unbiased segmentation and quantification of myofibers in skeletal muscle. Sci Rep.

[72] Patro R (2017). Salmon provides fast and bias-aware quantification of transcript expression. Nat Methods.

[73] Soneson C (2015). Differential analyses for RNA-seq: transcript-level estimates improve gene-level inferences. F1000Res.

[74] Love MI (2014). Moderated estimation of fold change and dispersion for RNA-seq data with DESeq2. Genome Biol.

