# Supplementary material for: Differential pathology and susceptibility to MBNL loss across muscles in myotonic dystrophy mouse models
Source: JCI Insight. 2025 Aug 14;10(18):e195836. doi: 10.1172/jci.insight.195836 (PMC12487862; doi:10.1172/jci.insight.195836)
Supplement: Supplemental data [file jciinsight-10-195836-s097.pdf]

Supplemental Figure 1.

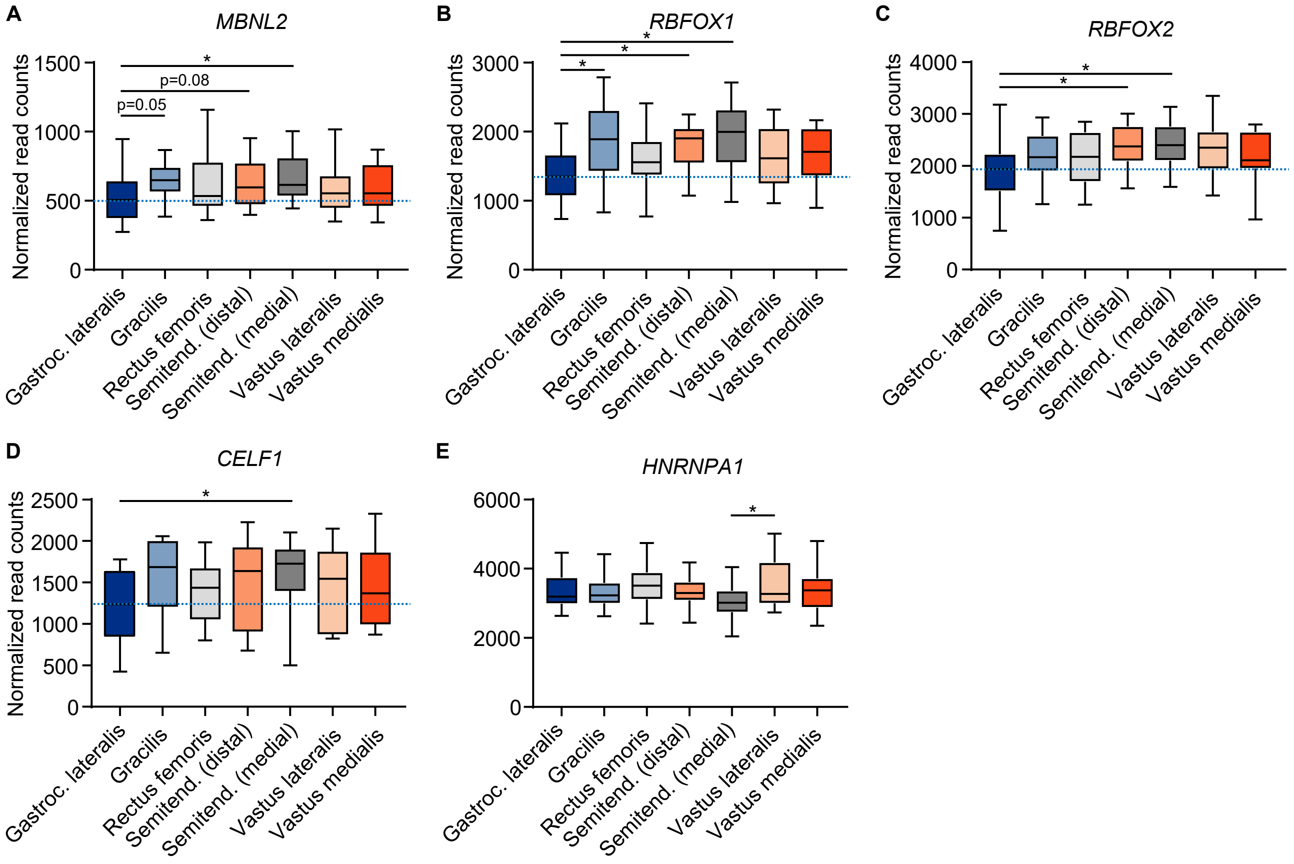

Supplemental Figure 1. Additional differential disease relevant gene expression across healthy human muscles. (A-E) Normalized read counts of RNAseq data from Abbassi-Daloii et al. (16) for (A) *MBNL2*, (B) *RBFOX1*, (C) *RBFOX2*, (D) *CELF1*, and (E) *HNRNPA1*. *, $P < 0.05$; One-way ANOVA with Tukey's multiple comparisons test. Dotted line indicates mean expression in the gastrocnemius lateralis, the only distal muscle biopsied for analysis. Paired samples from 20 healthy males 25±3.6 years old.

Supplemental Figure 2.

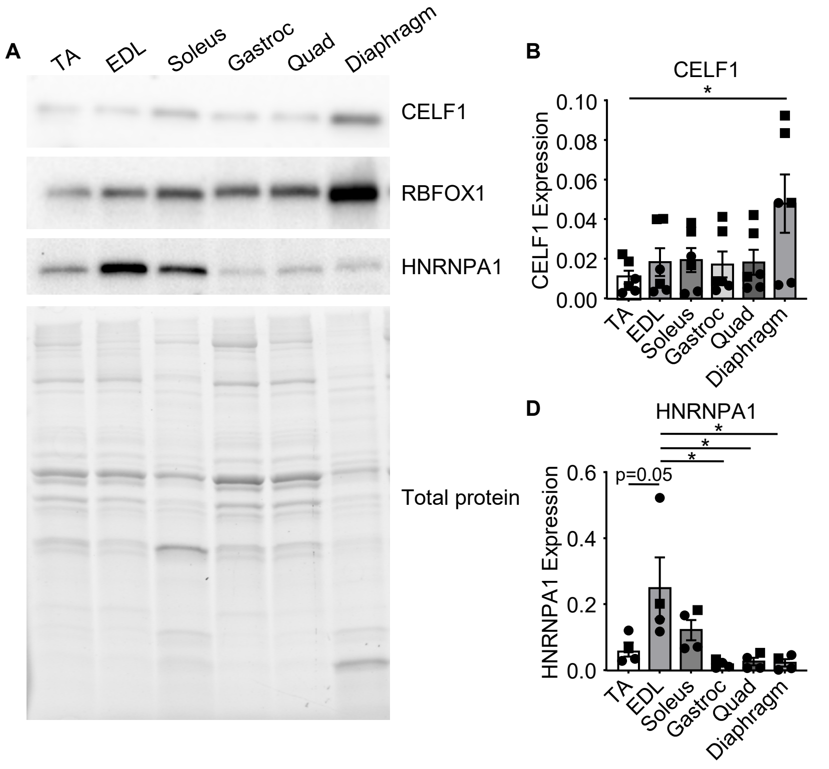

Supplemental Figure 2. Additional differential disease relevant protein expression across wild-type mouse muscles (A) Western blot for CELF1, RBFOX1, and HNRNPA1 in 8-12 week-old wild-type FVB mouse muscles. Data are representative of six independent experiments. (B-D) Quantification of CELF1 (B), RBFOX1 (C), and HNRNPA1 (D) western blots (Mean, error bars ± SEM: *, $P < 0.05$; One-way ANOVA with Tukey's multiple comparisons test). Expression normalized to total protein. Squares indicate males and circles indicate females.

Supplemental Figure 3.

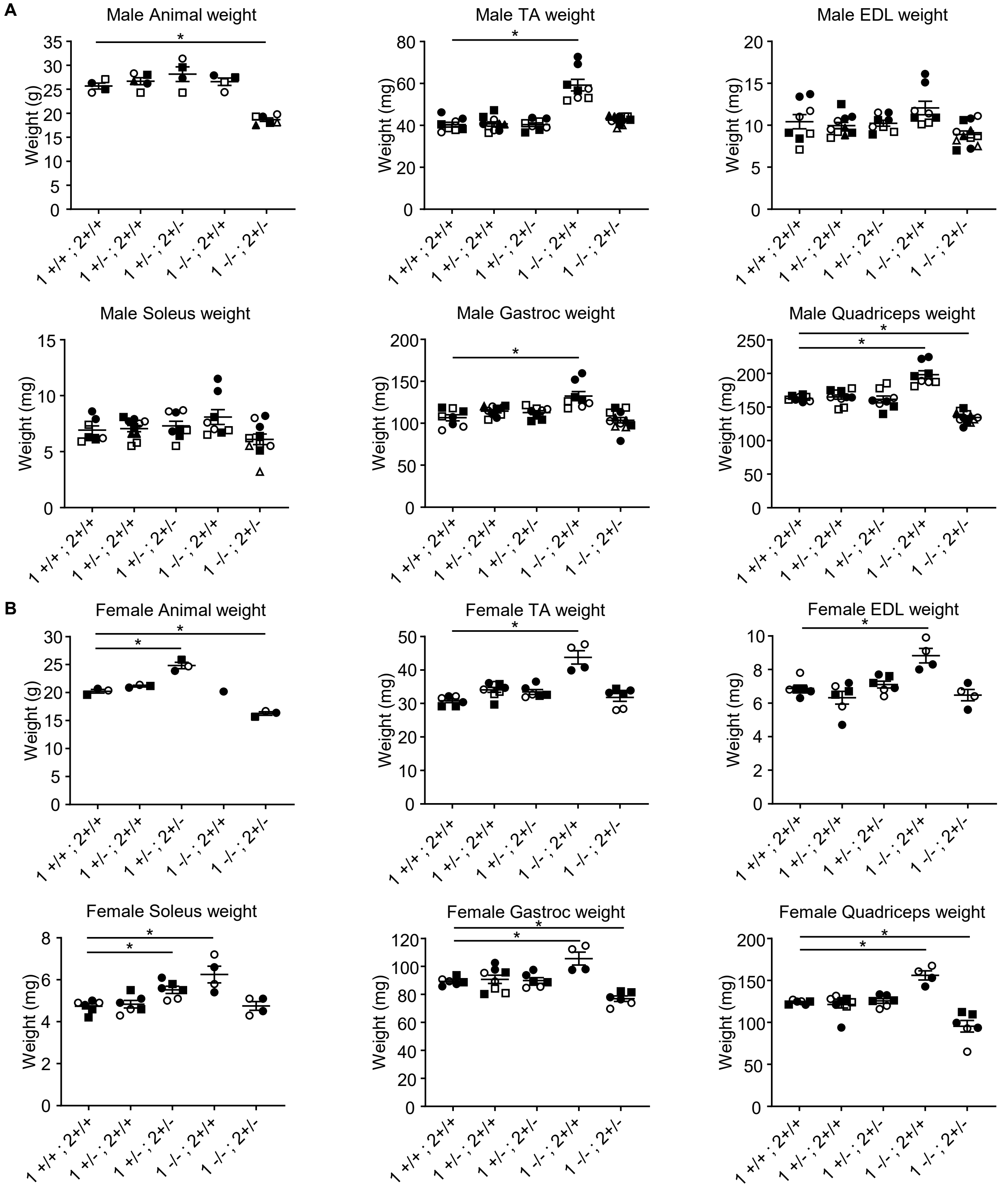

Supplemental Figure 3. Raw muscle weights. (A-B) Mean body and raw muscle weights from wild-type, *Mbnl1*$^{+/-}$, *Mbnl1*$^{+/-}$; *Mbnl2*$^{+/-}$, *Mbnl1*$^{-/-}$, and *Mbnl1*$^{-/-}$; *Mbnl2*$^{+/-}$ (A) male and (B) female mice. Error bars ± SEM: *, $P < 0.05$; One-way ANOVA with Dunnett's multiple comparisons test. Distinct shapes indicate different animals. Consistent filled or unfilled shapes indicate muscles from the same animal. n = 2-6 animals per genotype.

Supplemental Figure 4.

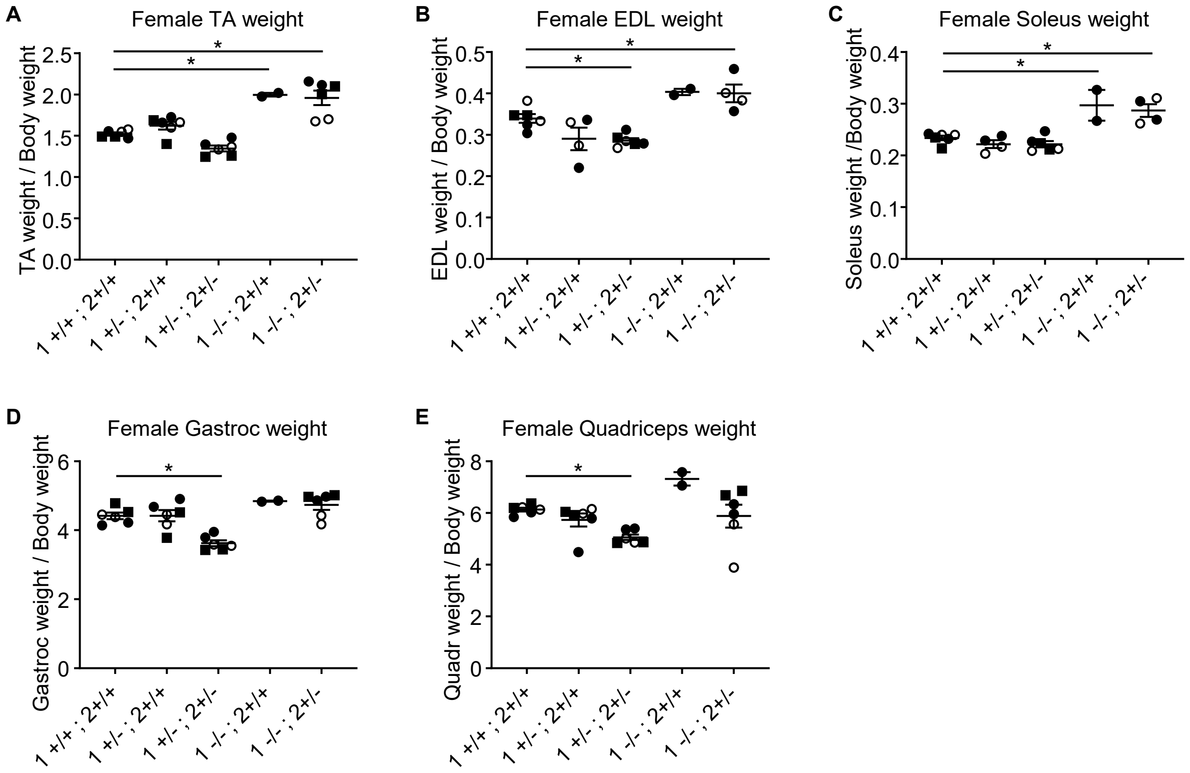

Supplemental Figure 4. Female normalized muscle weights. (A-E) Mean muscle weight normalized to body weight across an allelic series of 8-week-old female *Mbnl* KO mice (error bars ± SEM: *, P < 0.05; One-way ANOVA with Dunnett's multiple comparisons test). Distinct shapes indicate different animals. Consistent filled or unfilled shapes indicate muscles from the same animal.

Supplemental Figure 5.

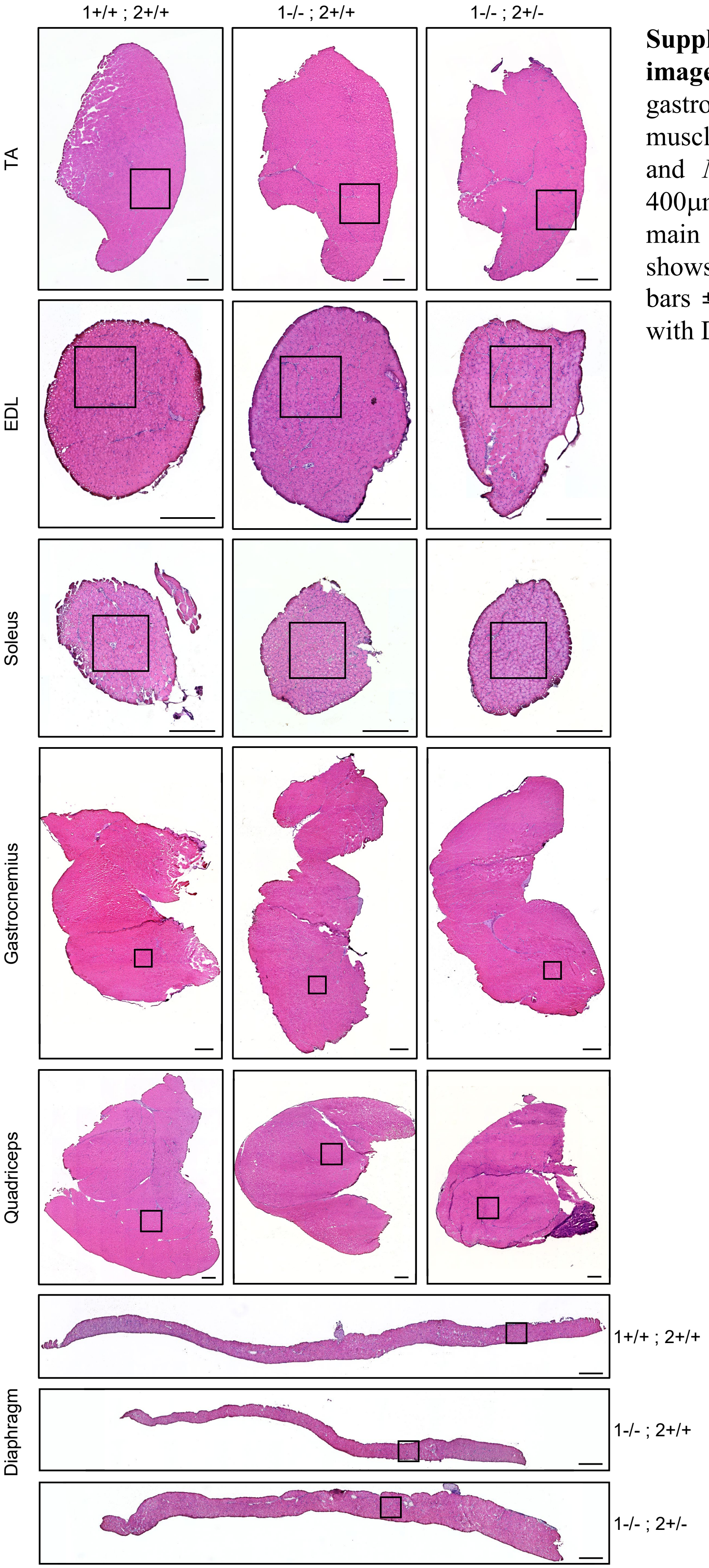

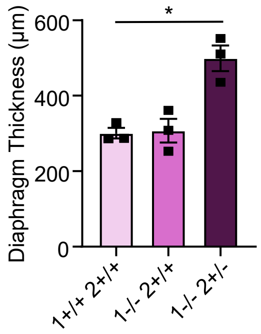

Supplemental Figure 5. Full size H&E images. H&E staining of TA, EDL, soleus, gastrocnemius, quadriceps, and diaphragm muscles from 8-week-old wild-type, *Mbnl1*$^{-/-}$, and *Mbnl1*$^{-/-}$; *Mbnl2*$^{+/-}$ mice. Scale bar = 400μm. Boxes indicate regions shown in main text. n=4 mice per genotype. Graph shows average diaphragm thickness (error bars ± SEM: *, $P < 0.05$; One-way ANOVA with Dunnett’s multiple comparisons test).

Supplemental Figure 6.

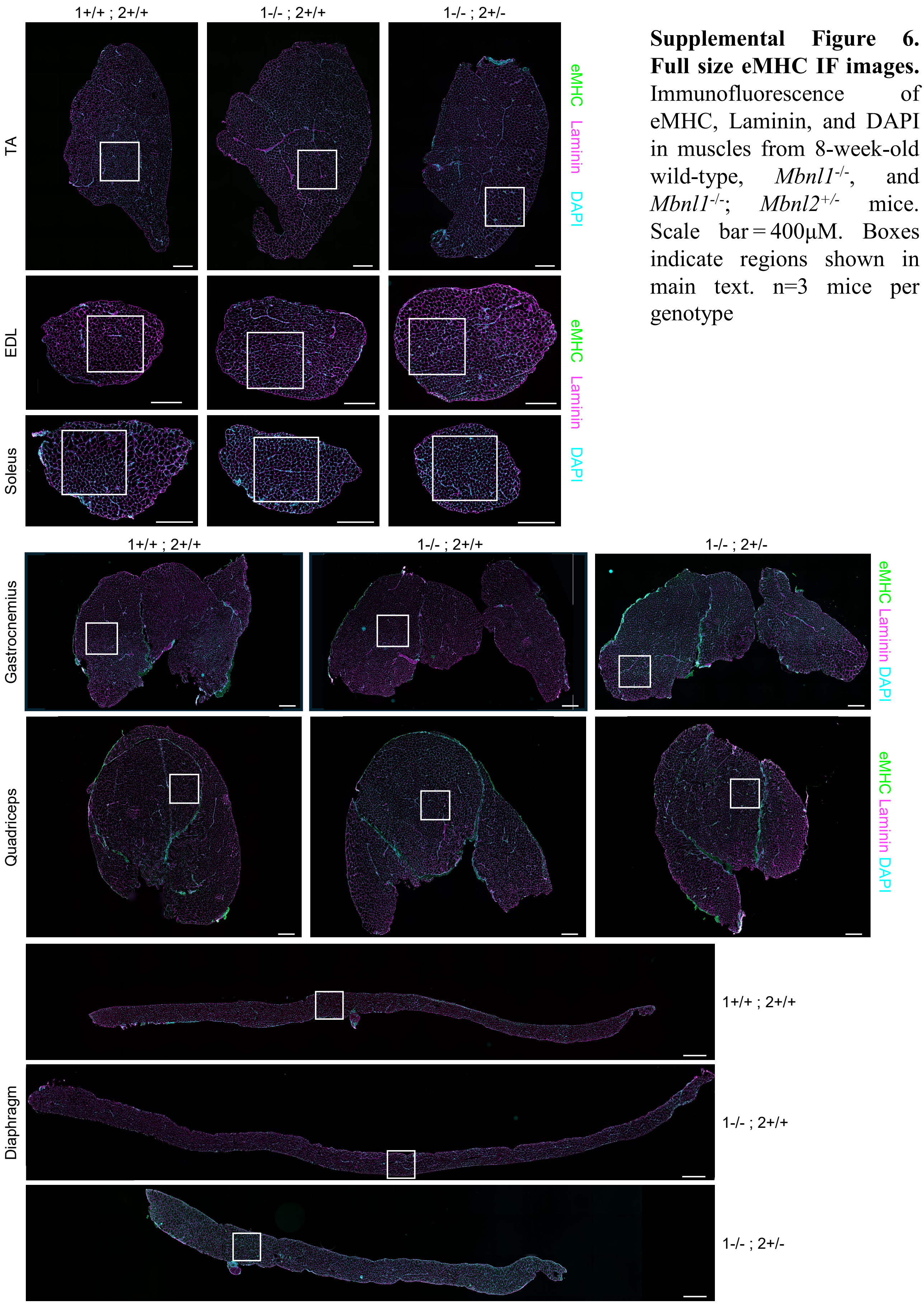

Supplemental Figure 6. Full size eMHC IF images. Immunofluorescence of eMHC, Laminin, and DAPI in muscles from 8-week-old wild-type, *Mbnl1*$^{-/-}$, and *Mbnl1*$^{-/-}$; *Mbnl2*$^{+/-}$ mice. Scale bar = 400μM. Boxes indicate regions shown in main text. n=3 mice per genotype

Supplemental Figure 7.

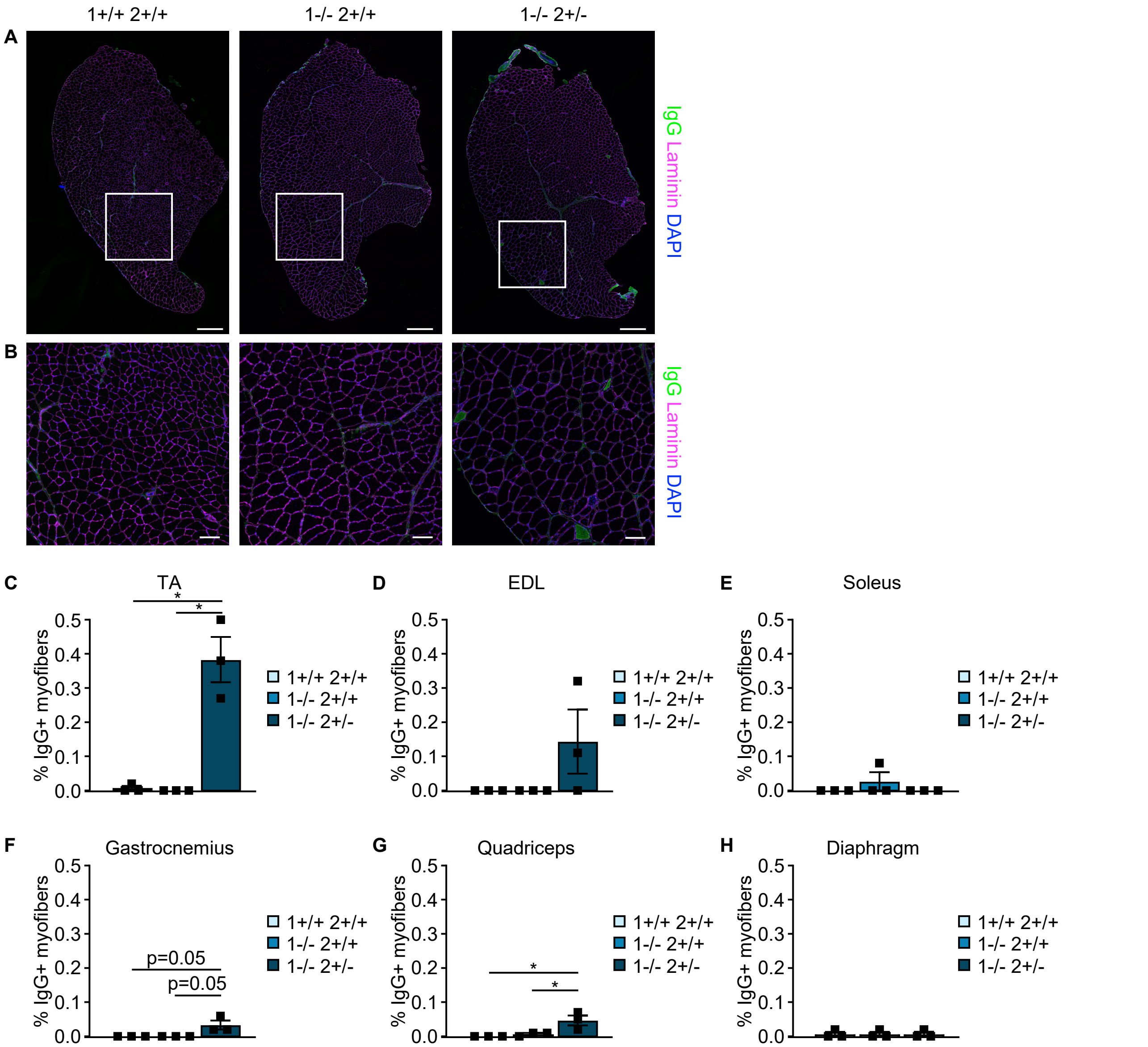

Supplemental Figure 7. Necrotic myofiber staining. (A-B) Immunofluorescence of mouse IgG, Laminin, and DAPI in TA muscles from 8-week-old wild-type, *Mbnl1*$^{-/-}$, and *Mbnl1*$^{-/-}$; *Mbnl2*$^{+/-}$ mice. (A) Scale bar = 400µM. Boxes indicate regions shown in (B). (B) Scale bar = 100µM. (C-H) Quantification of percent IgG+ myofibers per cross-section (error bars ± SEM: *, $p < 0.05$, One-way ANOVA with Tukey's multiple comparisons test). n=3 mice per genotype.

Supplemental Figure 8.

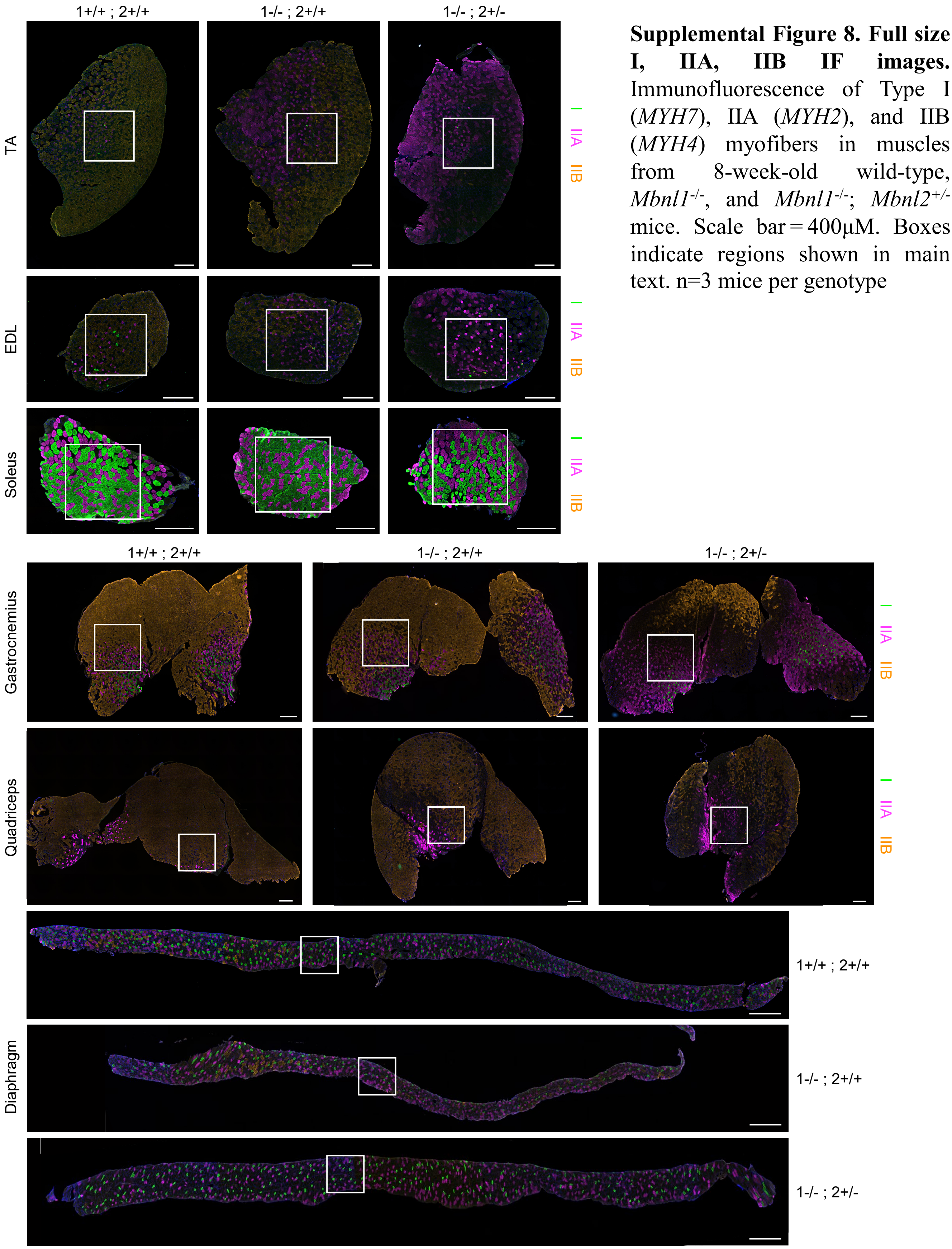

Supplemental Figure 8. Full size I, IIA, IIB IF images. Immunofluorescence of Type I (*MYH7*), IIA (*MYH2*), and IIB (*MYH4*) myofibers in muscles from 8-week-old wild-type, *Mbnl1*$^{-/-}$, and *Mbnl1*$^{-/-}$; *Mbnl2*$^{+/-}$ mice. Scale bar = 400µM. Boxes indicate regions shown in main text. n=3 mice per genotype

Supplemental Figure 9.

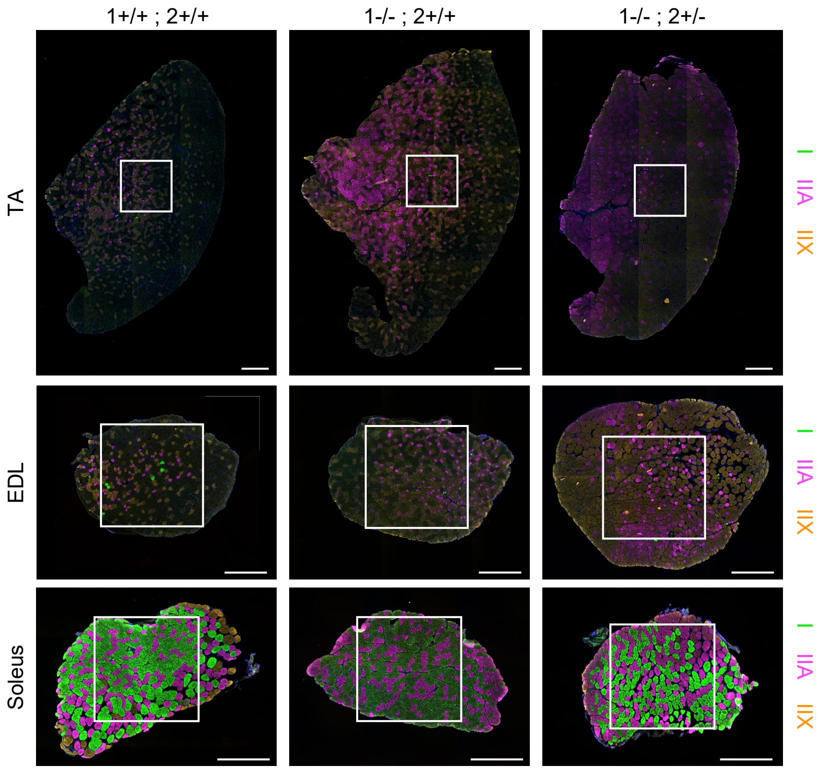

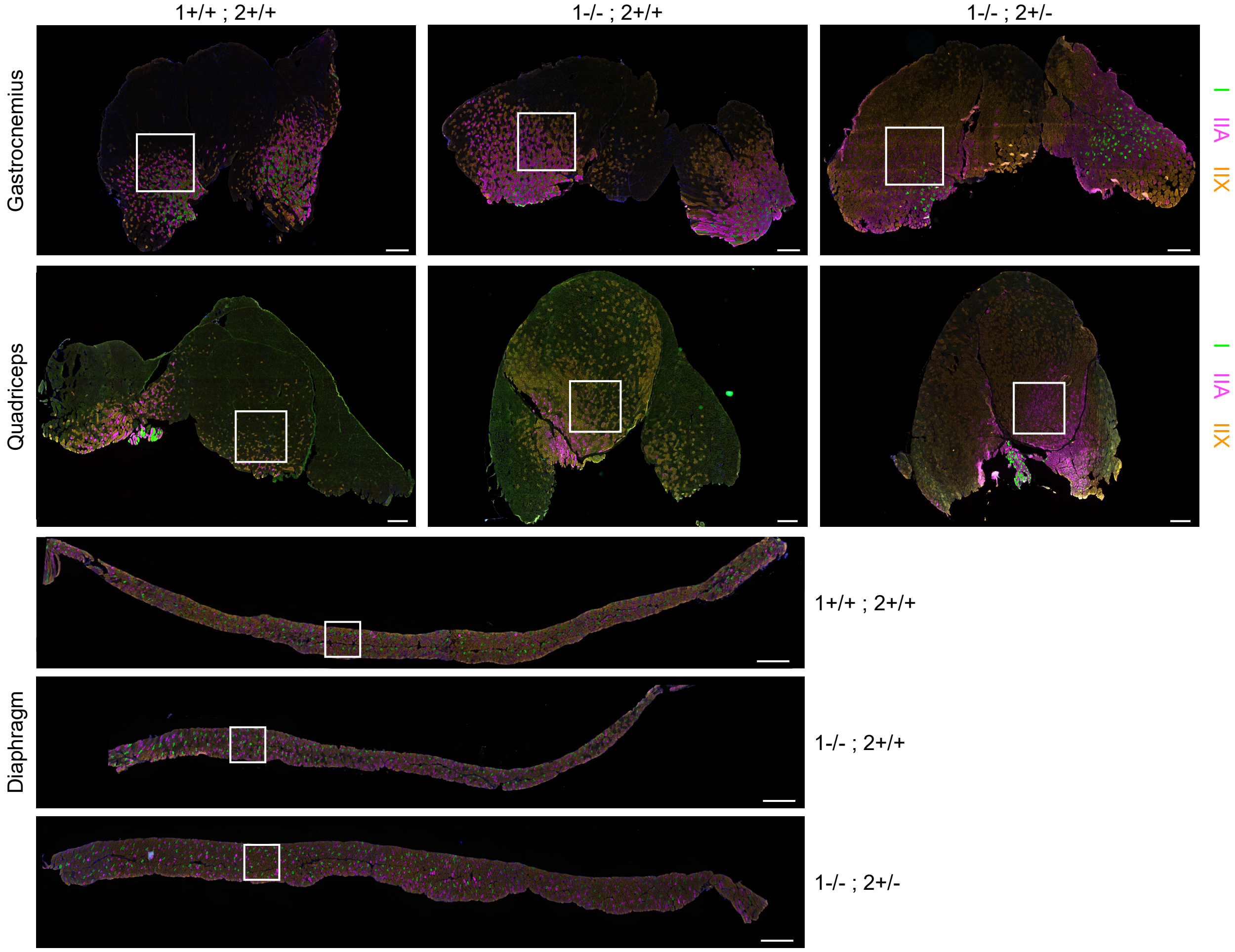

Supplemental Figure 9. Full size I, IIA, IIX IF images. Immunofluorescence of Type I (*MYH7*), IIA (*MYH2*), and IIX (*MYH1*) myofibers in muscles from 8-week-old wild-type, *Mbnl1*$^{-/-}$, and *Mbnl1*$^{-/-}$; *Mbnl2*$^{+/-}$ mice. Scale bar = 400μM. Boxes indicate regions shown in Fig. S10. IIB and IIX fibers cannot be stained simultaneously and thus were stained on serial sections. n=3 mice per genotype

Supplemental Figure 10.

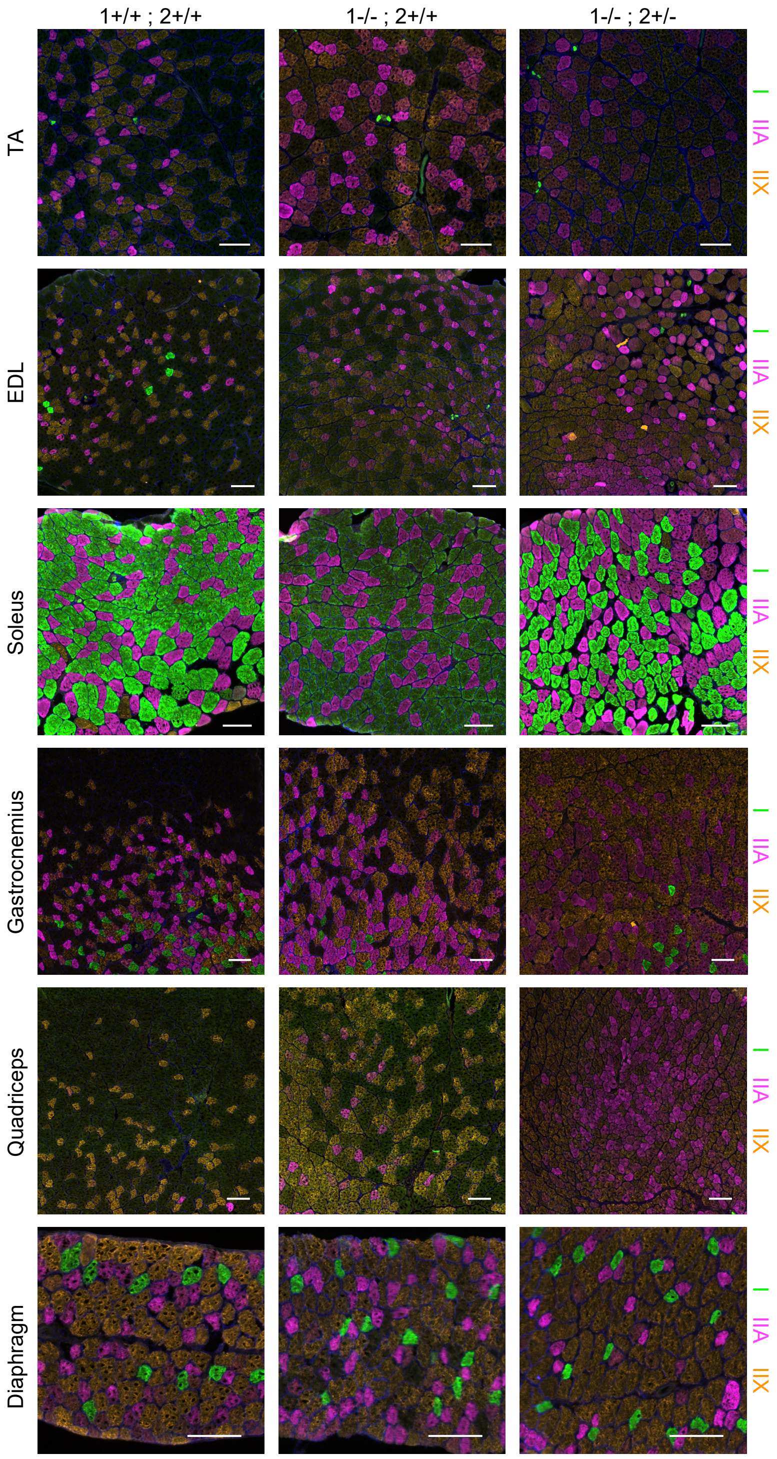

Supplemental Figure 10. Cropped I, IIA, IIX images. Zoomed in images of immunofluorescence from Figure S9 of Type I (*MYH7*), IIA (*MYH2*), and IIX (*MYH1*) myofibers in muscles from 8-week-old wild-type, *Mbnl1*$^{-/-}$, and *Mbnl1*$^{-/-}$; *Mbnl2*$^{+/-}$ mice. Scale bar = 100µM.

Supplemental Figure 11. Type I fiber size distribution

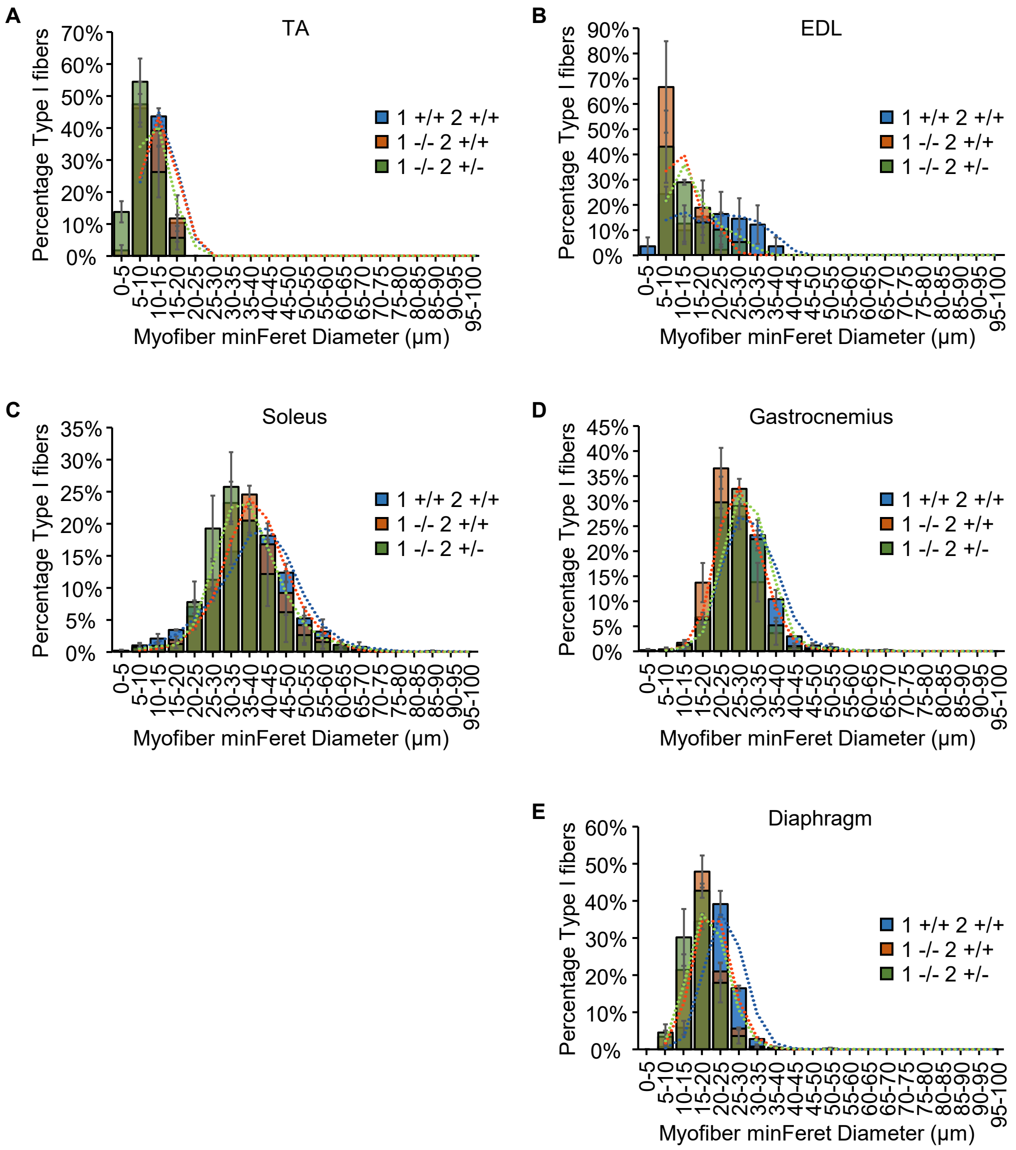

Supplemental Figure 11. Type I Myofiber size distribution. (A-E) Myofiber size distribution of Type I fibers by MinFeret diameter of the indicated muscles from wild-type, *Mbnl1*$^{-/-}$, and *Mbnl1*$^{-/-}$; *Mbnl2*$^{+/-}$ mice (error bars ± SEM). Bars are overlapping. n = 3 male mice per genotype.

Supplemental Figure 12. Type IIA fiber size distribution

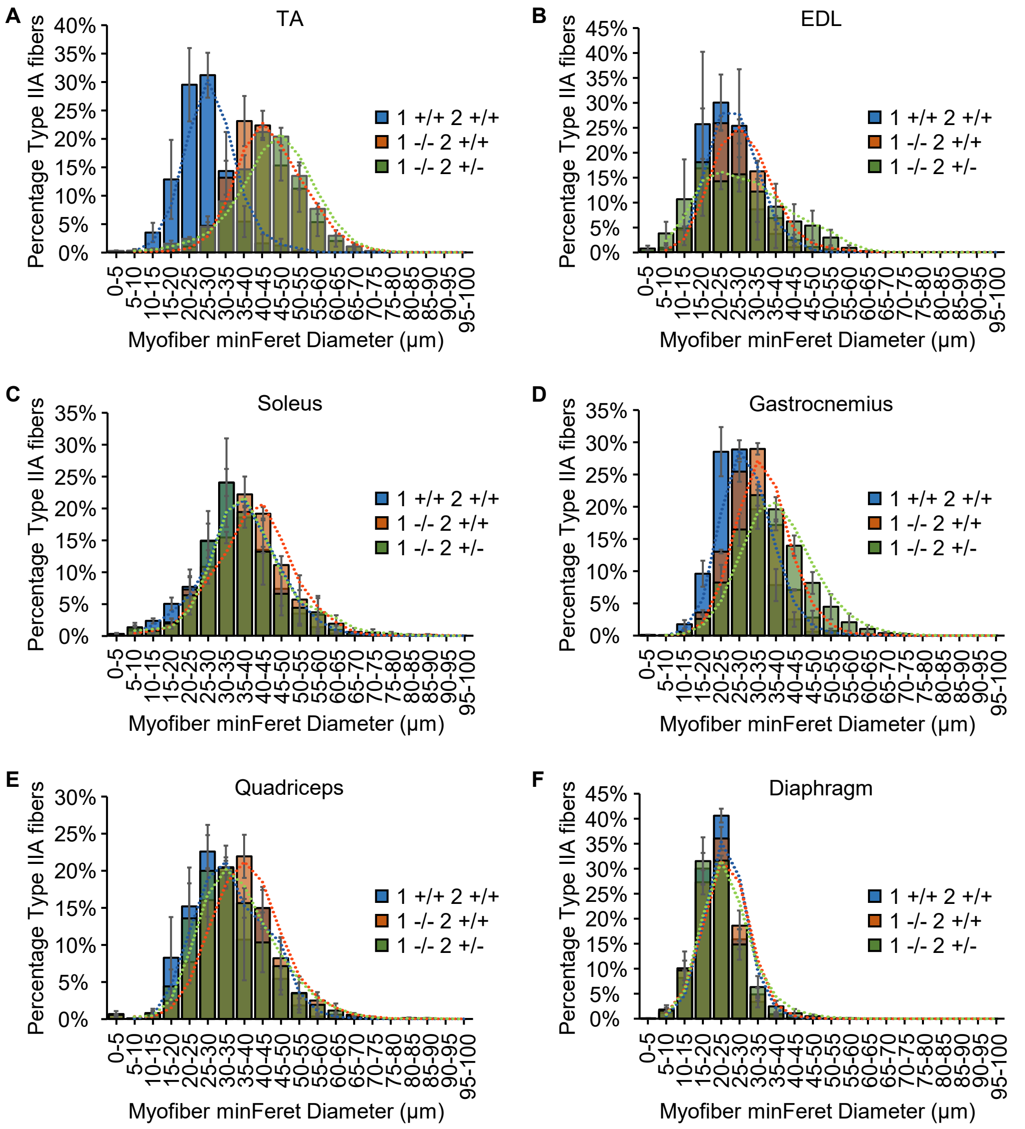

Supplemental Figure 12. Type IIA Myofiber size distribution. (A-F) Myofiber size distribution of Type IIA fibers by MinFeret diameter of the indicated muscles from wild-type, *Mbnl1*$^{-/-}$, and *Mbnl1*$^{-/-}$; *Mbnl2*$^{+/-}$ mice (error bars ± SEM). Bars are overlapping. n = 3 male mice per genotype.

Supplemental Figure 13. Type IIX fiber size distribution

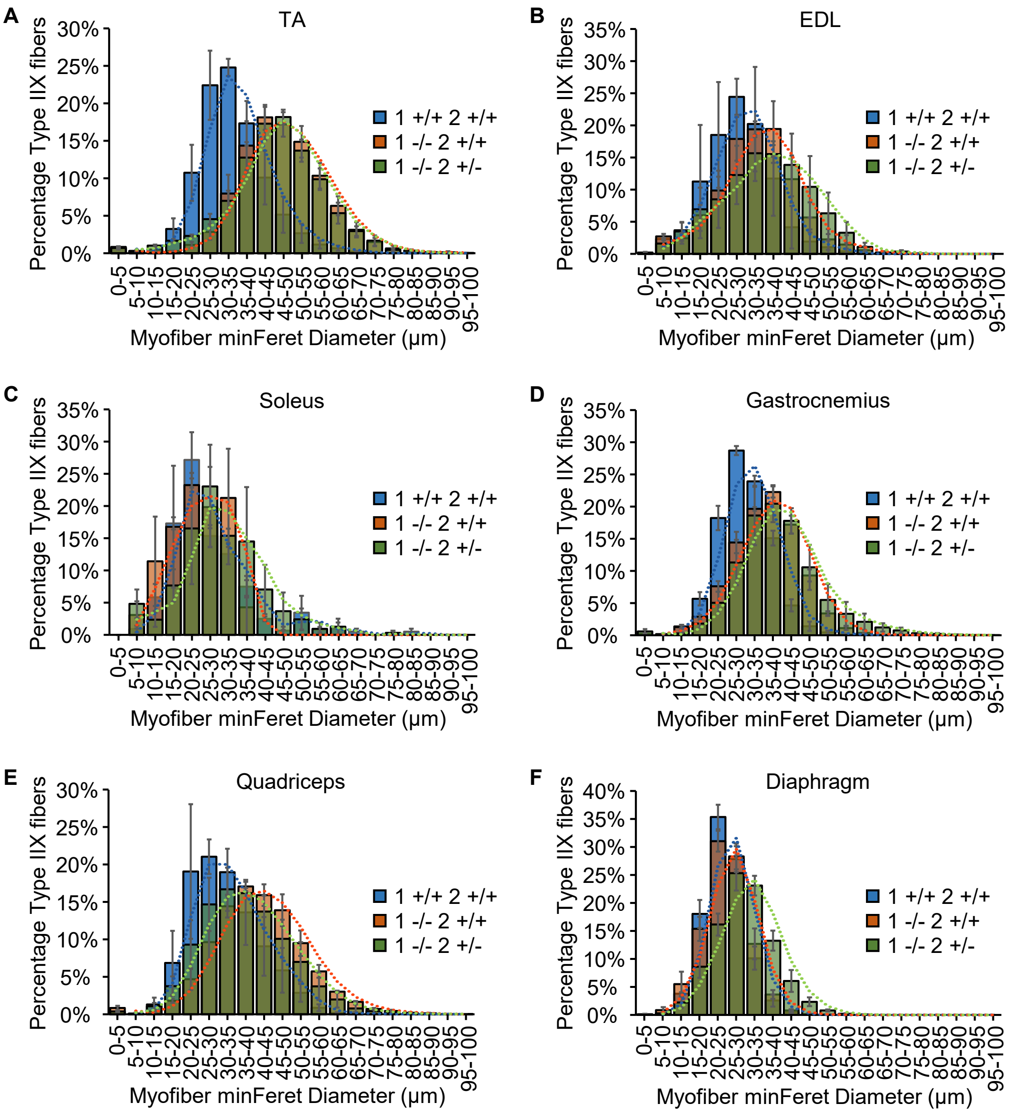

Supplemental Figure 13. Type IIX Myofiber size distribution. (A-F) Myofiber size distribution of Type IIX fibers by MinFeret diameter of the indicated muscles from wild-type, *Mbnl1*$^{-/-}$, and *Mbnl1*$^{-/-}$; *Mbnl2*$^{+/-}$ mice (error bars ± SEM). Bars are overlapping. n = 3 male mice per genotype.

Supplemental Figure 14. Type IIB fiber size distribution

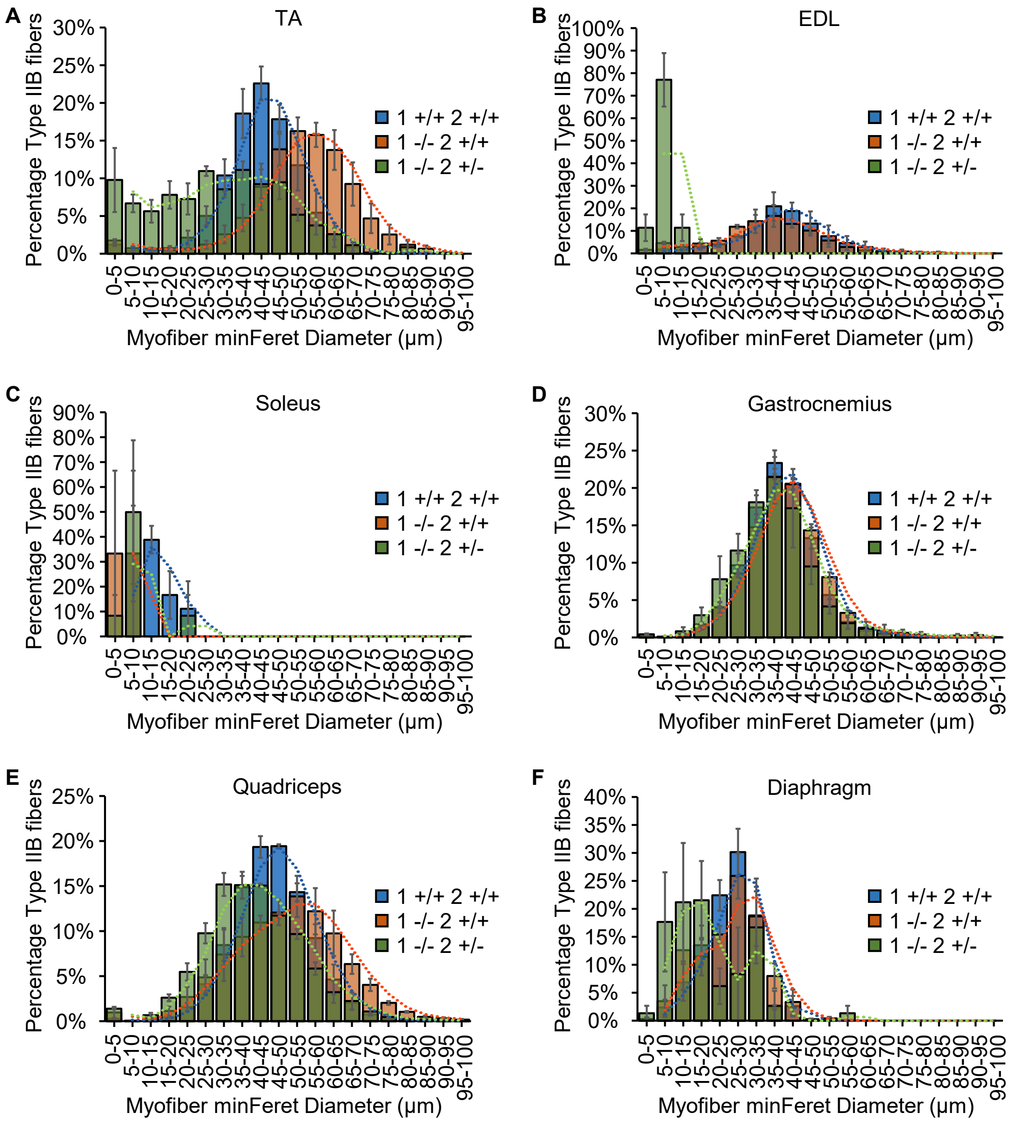

Supplemental Figure 14. Type IIB Myofiber size distribution. (A-F) Myofiber size distribution of Type IIB fibers by MinFeret diameter of the indicated muscles from wild-type, *Mbnl1*$^{-/-}$, and *Mbnl1*$^{-/-}$; *Mbnl2*$^{+/-}$ mice (error bars ± SEM). Bars are overlapping. n = 3 male mice per genotype.

Supplemental Figure 15.

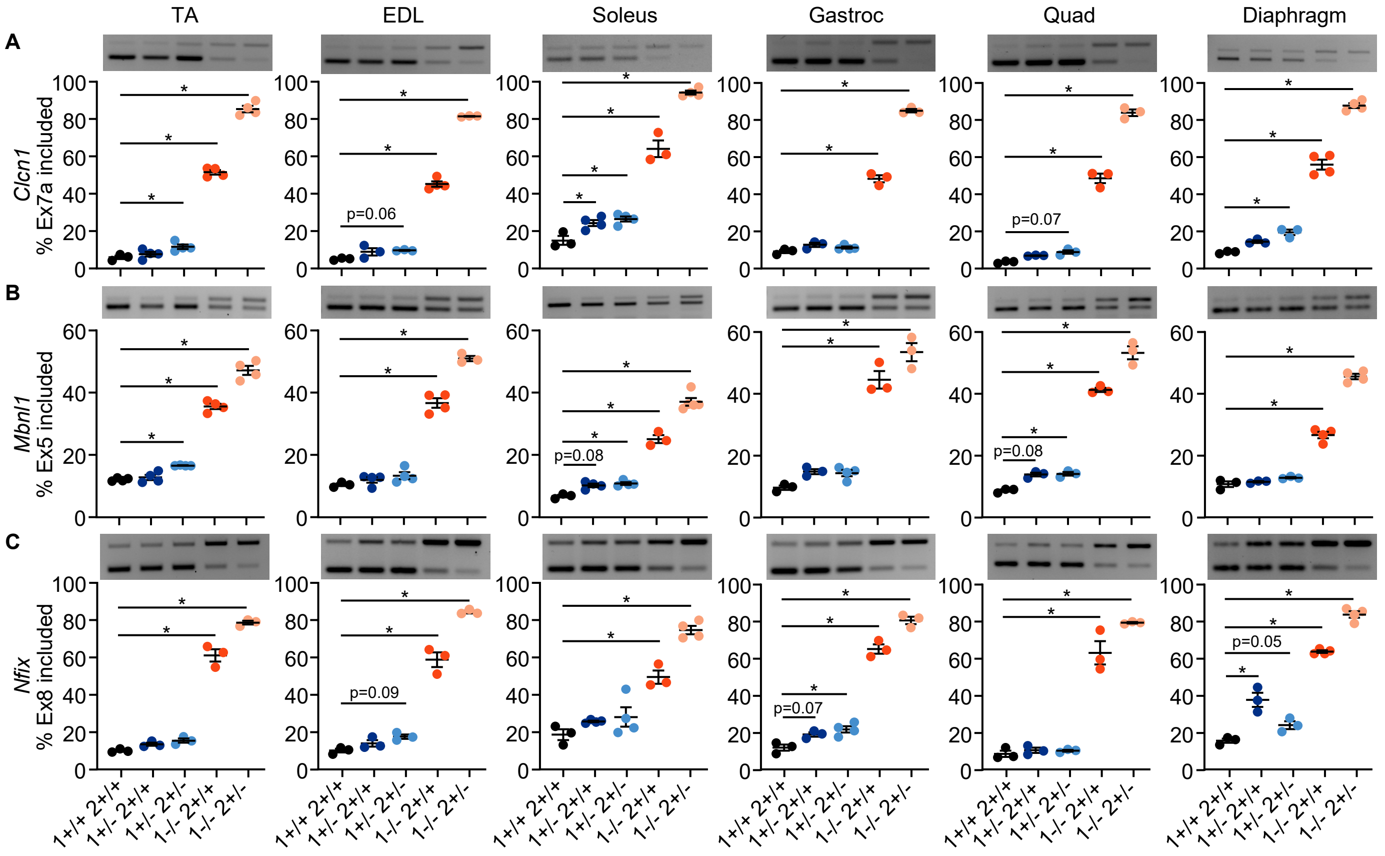

Supplemental Figure 15. Additional splicing events. (A-C) RT-PCR of (A) *Clcn1* exon 7a, (B) *Mbnl1* exon 5, and (C) *Nfix* exon 8 alternative splicing in 8wk old mouse muscles with genetic titration of MBNL (error bars ± SEM: *, $p < 0.05$, One-way ANOVA with Dunnett's multiple comparison's test). n=3-4 mice per genotype.
