# Supplementary material for: Differential pathology and susceptibility to MBNL loss across muscles in myotonic dystrophy mouse models
Source: JCI Insight. 2025 Aug 14;10(18):e195836. doi: 10.1172/jci.insight.195836 (PMC12487862; doi:10.1172/jci.insight.195836)

Figure 2D. MBNL1

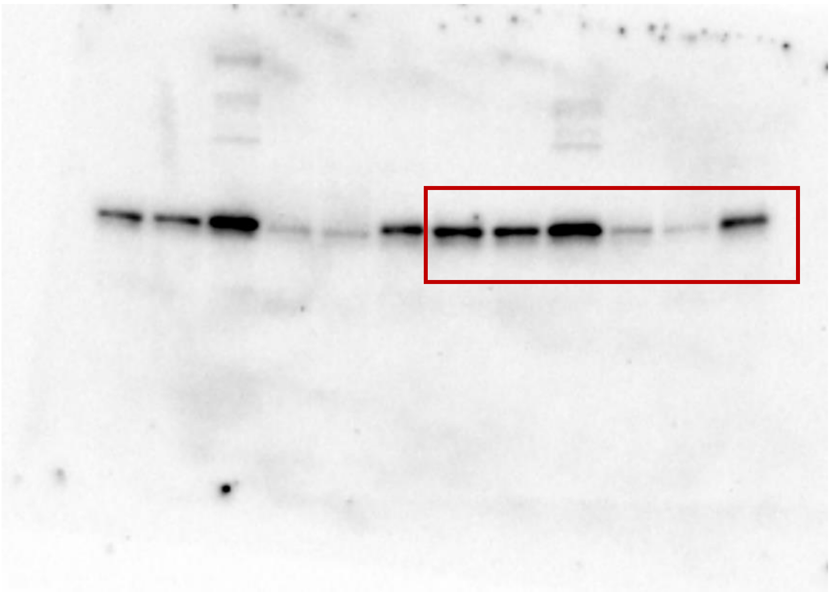

Figure 2D. Total protein

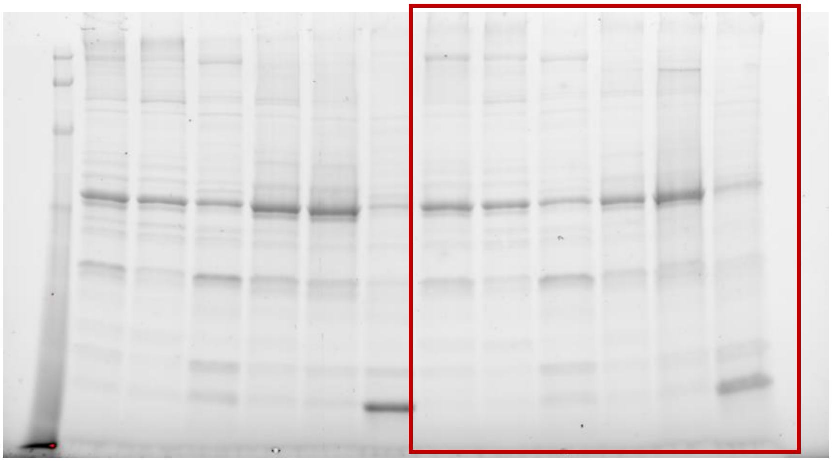

Figure S2A. CELF1

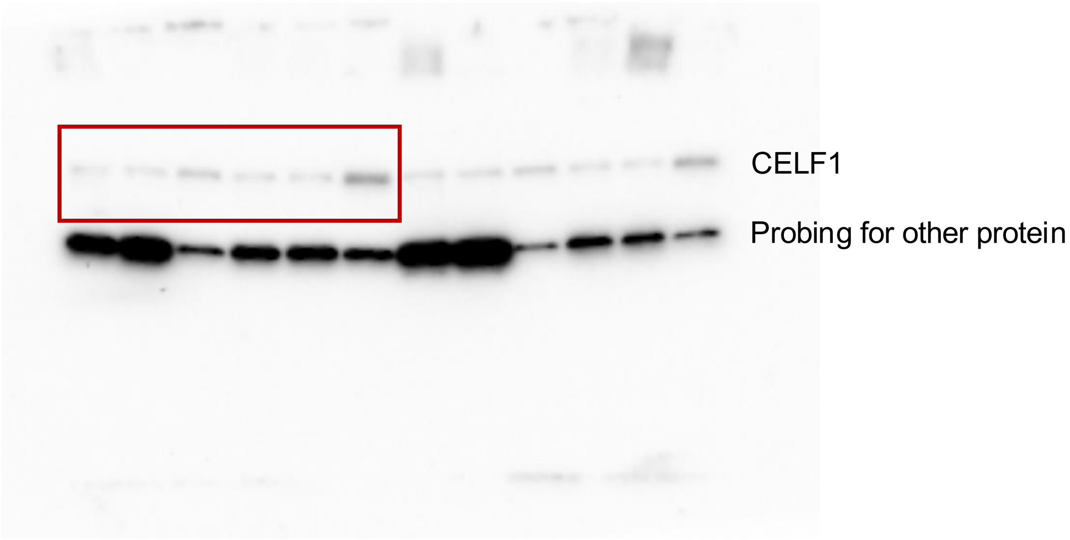

Figure S2A. Total protein

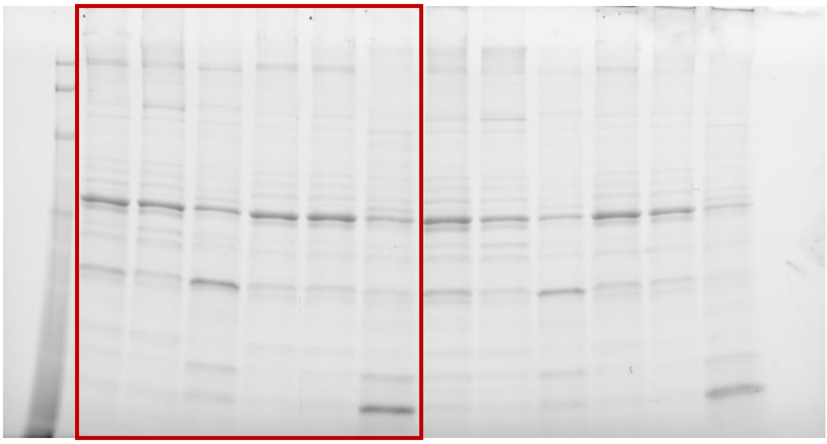

Figure S2A. RBFOX1

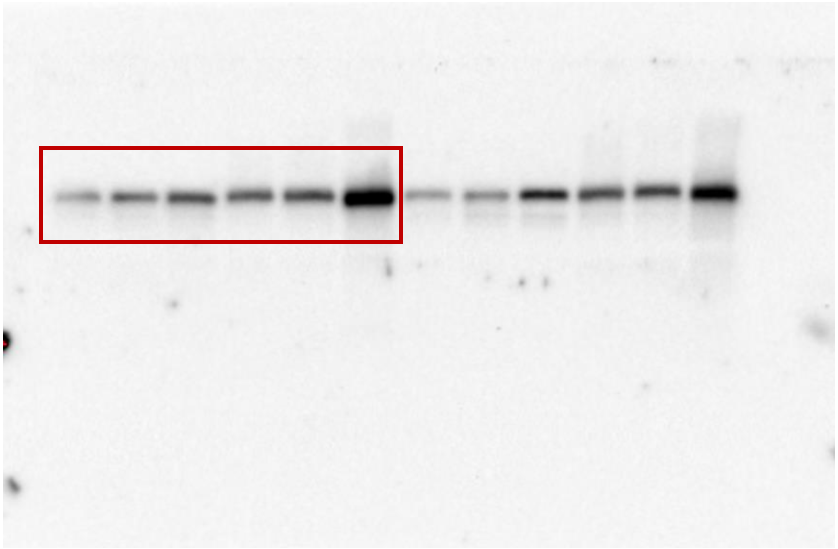

Figure S2A. Total protein

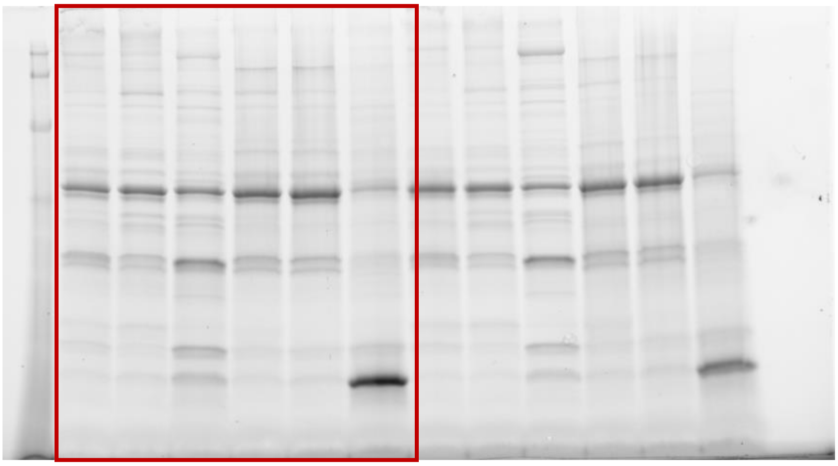

Figure S2A. HNRNPA1

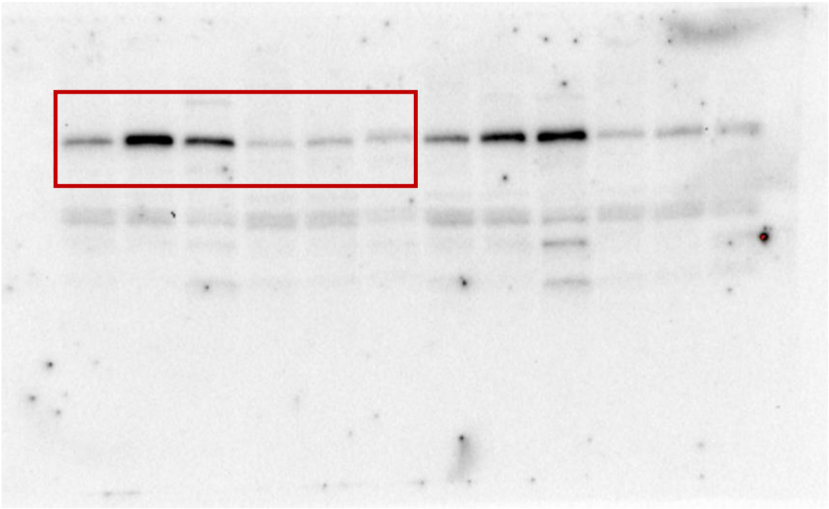

Figure S2A. Total protein

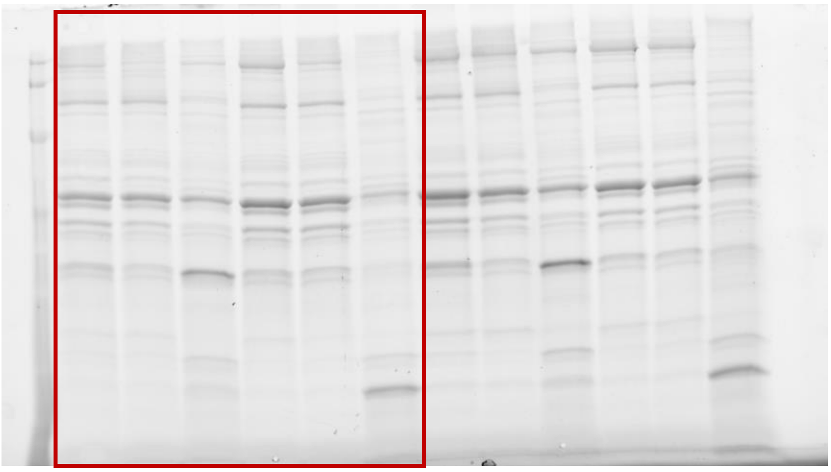

Figure 8A. ***Atp2a1***

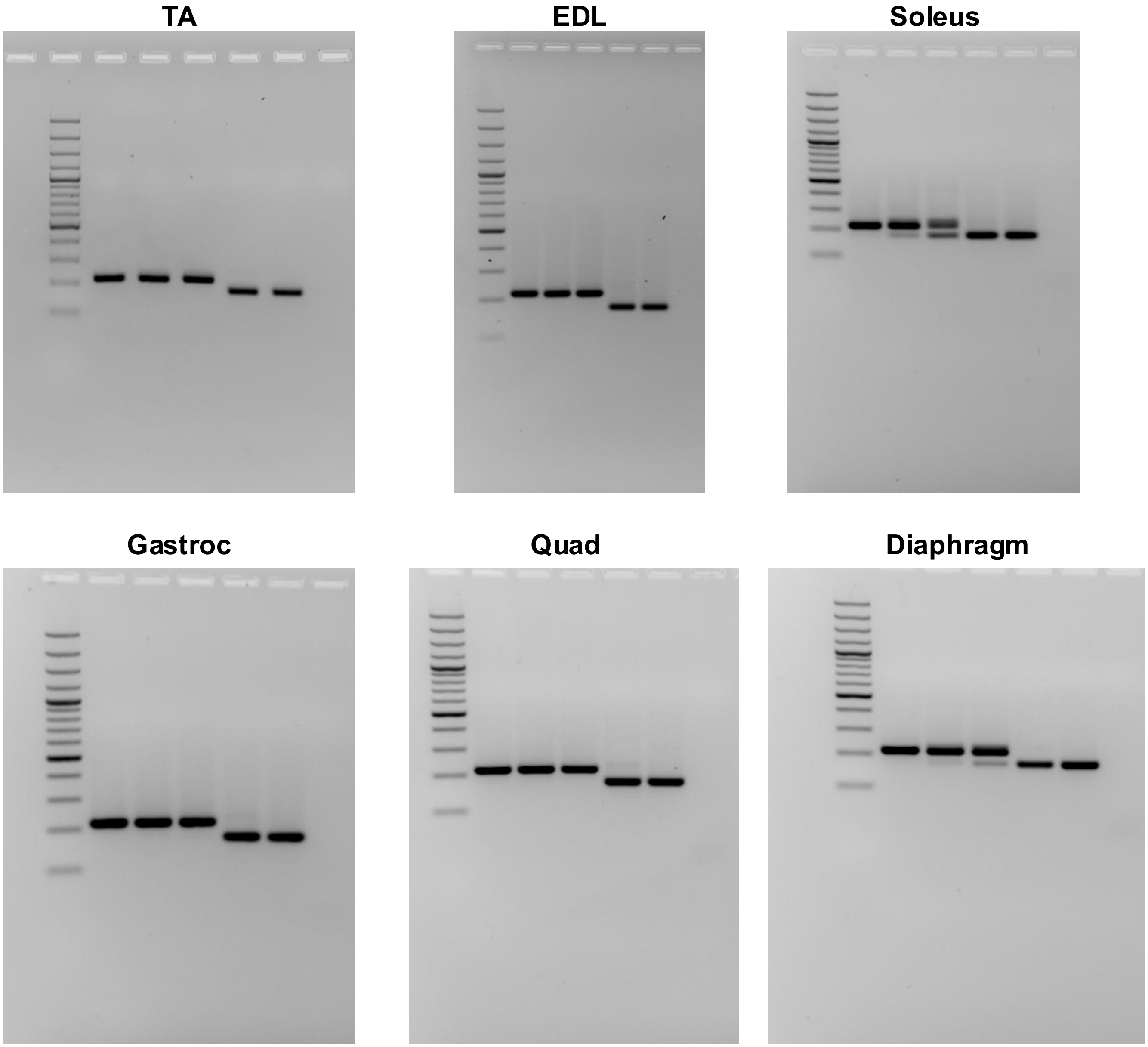

Figure 8B. *Cacna1s*

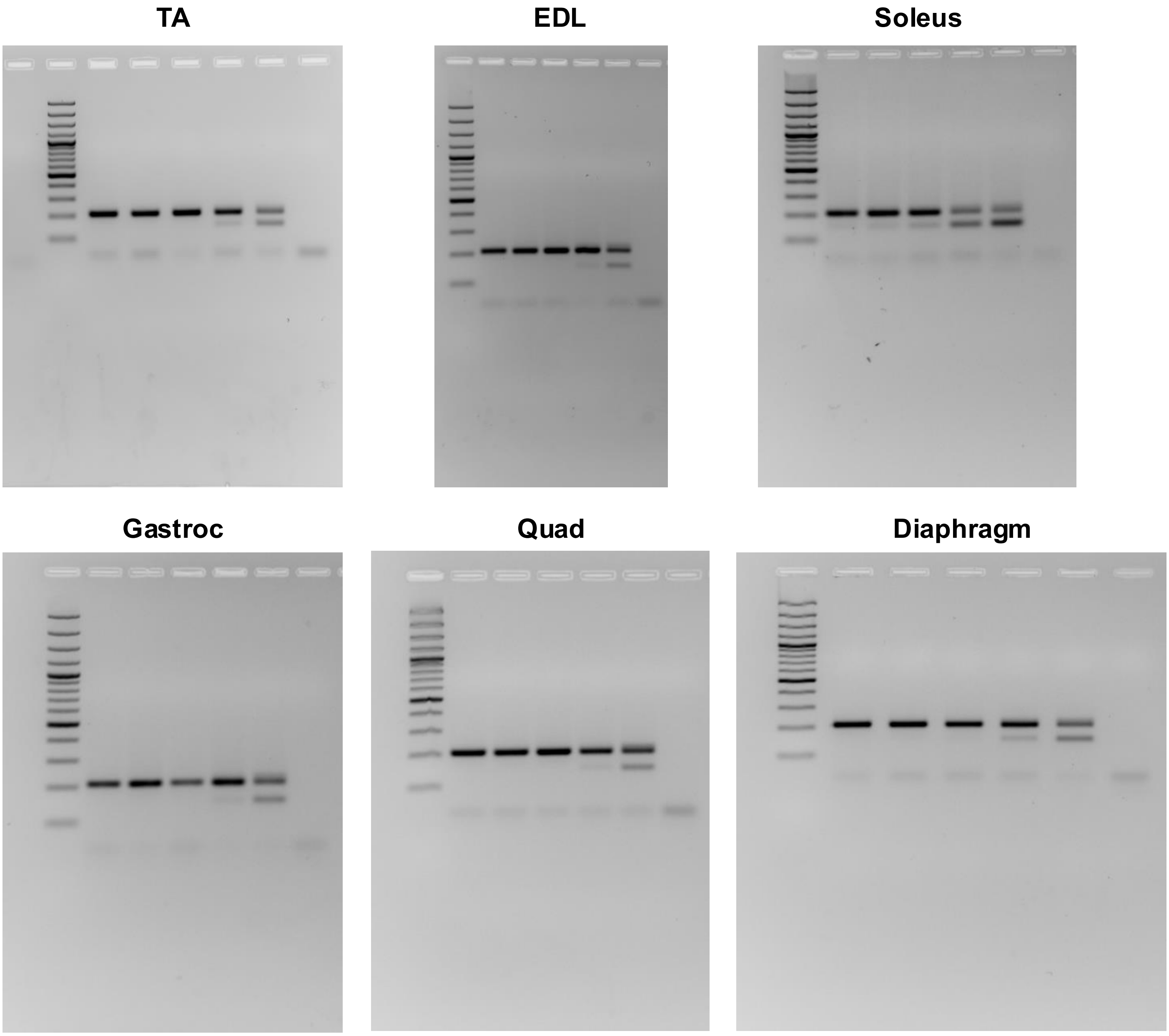

Figure 8C. *Bin1*

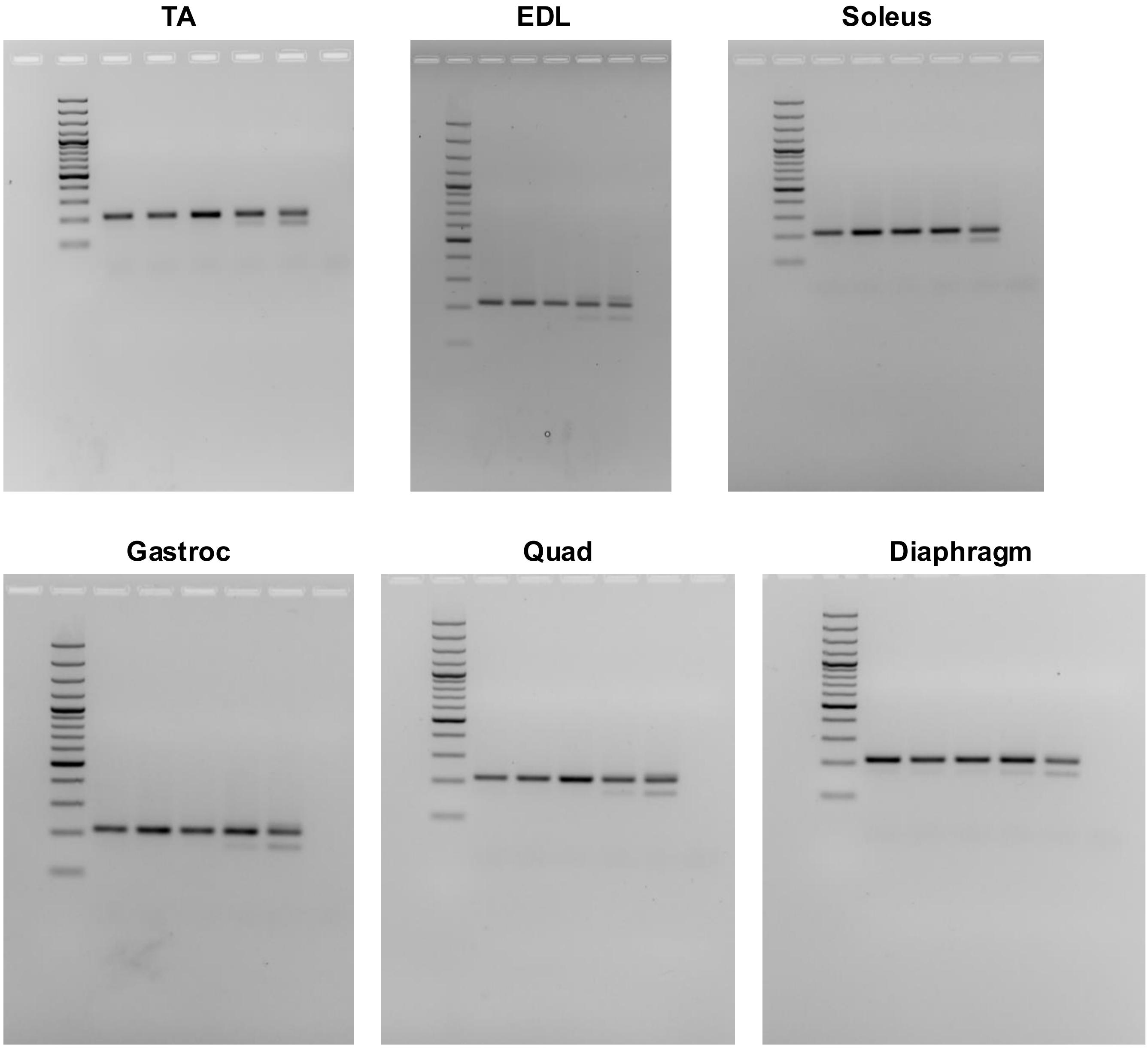

Figure 8D. ***Insr***

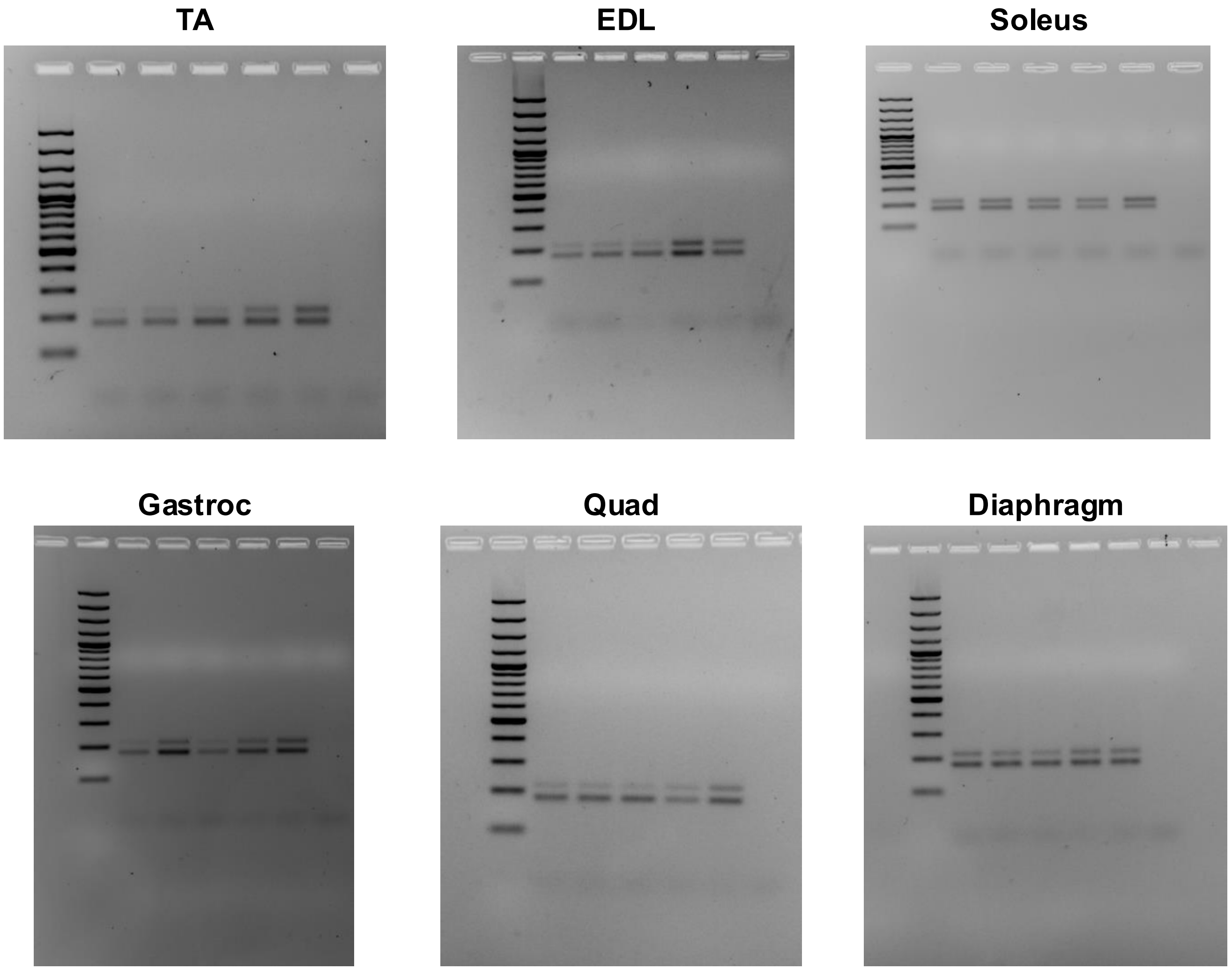

Figure S15A. *Clcn1*

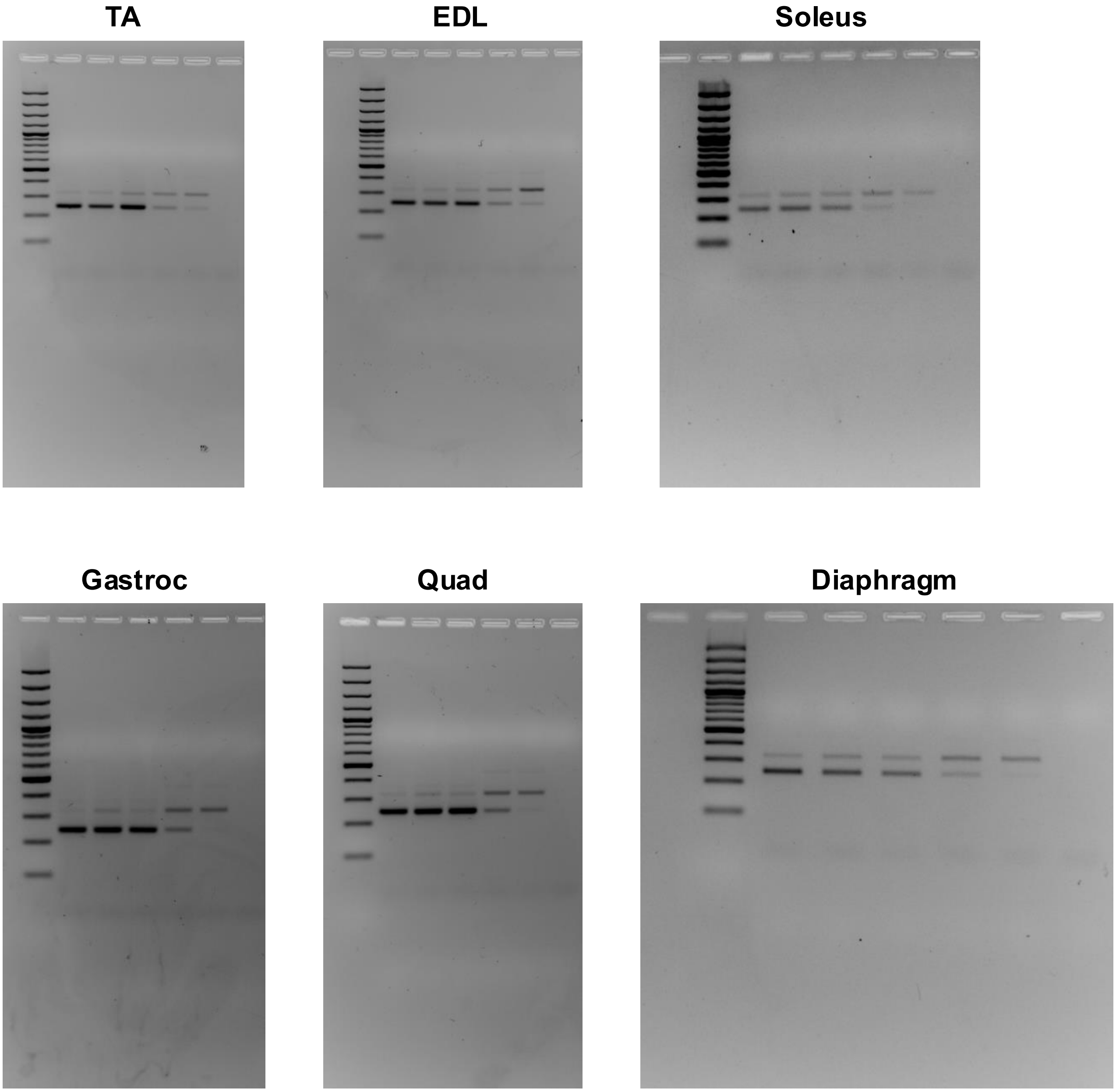

Figure S15B. ***Mbnl1***

TA

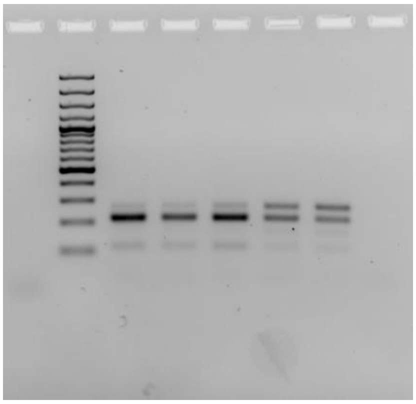

EDL

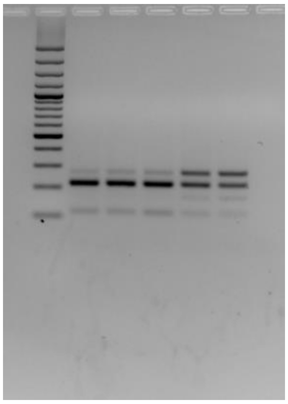

Soleus

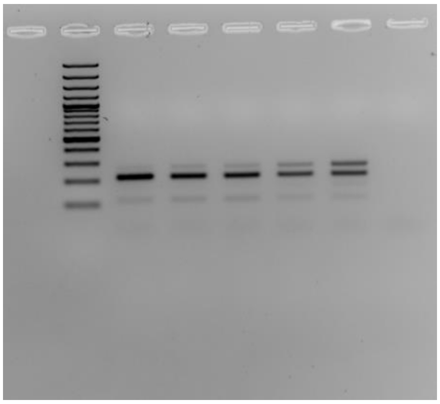

Gastroc

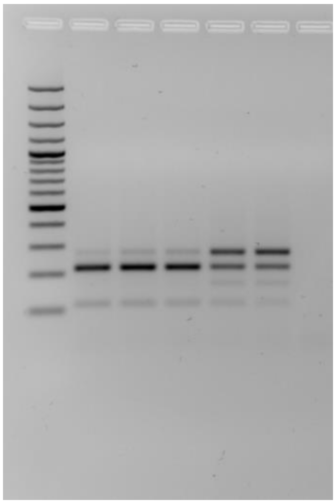

Quad

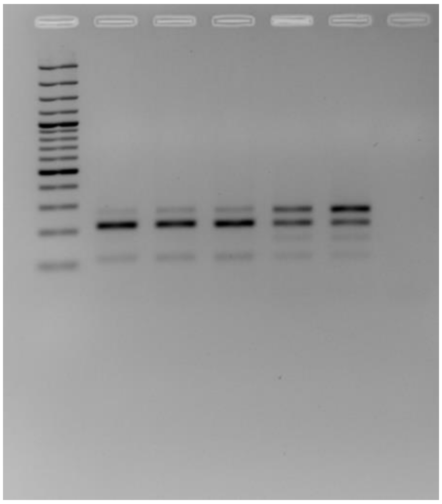

Diaphragm

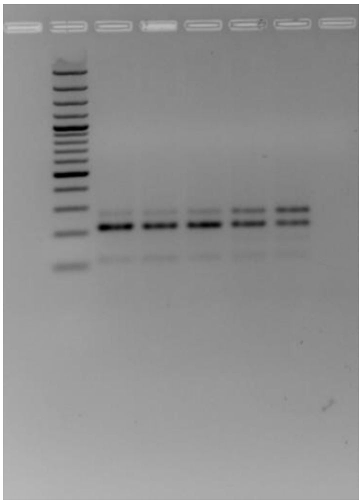

Figure S15C. *Nfix*

TA

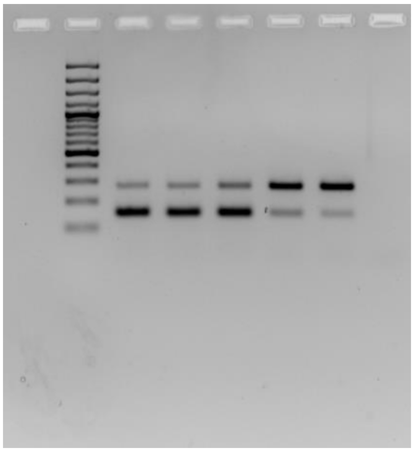

EDL

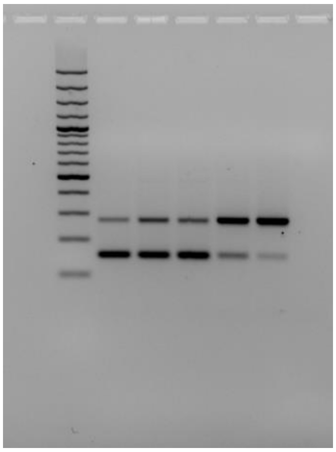

Soleus

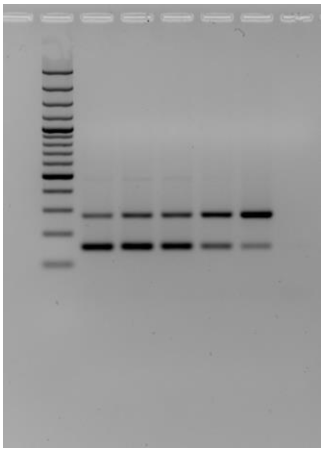

Gastroc

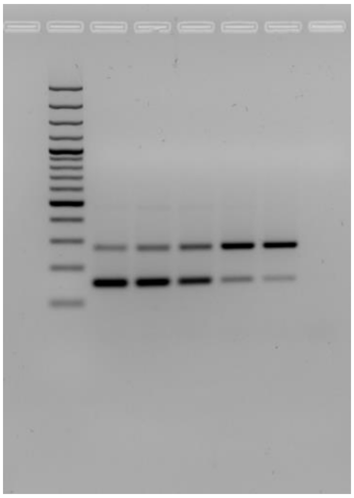

Quad

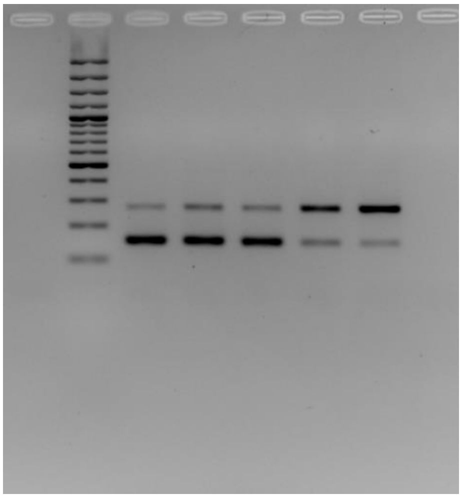

Diaphragm

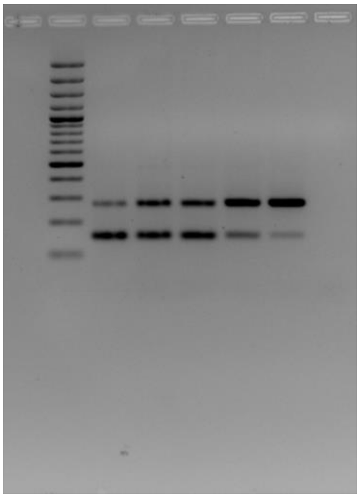

Supplement: Unedited blot and gel images [file jciinsight-10-195836-s098.pdf]
